# Western diet increases brain metabolism and adaptive immune responses in a mouse model of amyloidosis

**DOI:** 10.1186/s12974-024-03080-0

**Published:** 2024-05-14

**Authors:** Marilena Poxleitner, Sabrina H. L. Hoffmann, Georgy Berezhnoy, Tudor M. Ionescu, Irene Gonzalez-Menendez, Florian C. Maier, Dominik Seyfried, Walter Ehrlichmann, Leticia Quintanilla-Martinez, Andreas M. Schmid, Gerald Reischl, Christoph Trautwein, Andreas Maurer, Bernd J. Pichler, Kristina Herfert, Nicolas Beziere

**Affiliations:** 1 Werner Siemens Imaging Center, Department of Preclinical Imaging and Radiopharmacy, Eberhard Karls University Tübingen, Tübingen, Germany; 2 Department of Pathology and Neuropathology, University Hospital Tübingen, Eberhard Karls University, Tübingen, Germany; 3 Cluster of Excellence CMFI (EXC 2124) “Controlling Microbes to Fight Infections”, Eberhard Karls University, Tübingen, Germany; 4 Cluster of Excellence iFIT (EXC 2180) “Image Guided and Functionally Instructed Tumor Therapies”, Eberhard Karls University, Tübingen, Germany

**Keywords:** Western diet, Alzheimer’s disease, PET imaging, [^18^F]FDG, [^18^F]FTHA, [^18^F]GE-180, APPPS1, ^1^H spectroscopy, Metabolomics, Flow cytometry

## Abstract

Diet-induced increase in body weight is a growing health concern worldwide. Often accompanied by a low-grade metabolic inflammation that changes systemic functions, diet-induced alterations may contribute to neurodegenerative disorder progression as well. This study aims to non-invasively investigate diet-induced metabolic and inflammatory effects in the brain of an APPPS1 mouse model of Alzheimer’s disease. [^18^F]FDG, [^18^F]FTHA, and [^18^F]GE-180 were used for in vivo PET imaging in wild-type and APPPS1 mice. Ex vivo flow cytometry and histology in brains complemented the in vivo findings. ^1^H- magnetic resonance spectroscopy in the liver, plasma metabolomics and flow cytometry of the white adipose tissue were used to confirm metaflammatory condition in the periphery. We found disrupted glucose and fatty acid metabolism after Western diet consumption, with only small regional changes in glial-dependent neuroinflammation in the brains of APPPS1 mice. Further ex vivo investigations revealed cytotoxic T cell involvement in the brains of Western diet-fed mice and a disrupted plasma metabolome. ^1^H-magentic resonance spectroscopy and immunological results revealed diet-dependent inflammatory-like misbalance in livers and fatty tissue. Our multimodal imaging study highlights the role of the brain-liver-fat axis and the adaptive immune system in the disruption of brain homeostasis in amyloid models of Alzheimer’s disease.

**Supplementary Information:**

The online version contains supplementary material available at 10.1186/s12974-024-03080-0.

## Introduction

Overweight and obesity are serious health problems with an increasing prevalence worldwide [1]. In longitudinal studies mid-life obesity in overweight and obese individuals has been linked to changing lifestyles, characterized by reduced physical activity and poor dietary choices. These alterations result in metabolic disorders such as type 2 diabetes, and cardiovascular disease have been identified as risk factors for developing dementia and cognitive decline decades later [2–5]. Numerous investigations described the systemic alterations through high-caloric diets like Western diets (WDs) [6–8]. In response to obesity and associated chronic oversupply of fatty acids and sugar, a low-grade chronic inflammation develops, which if persisting over time, leads to a constant release of inflammatory effectors into the periphery [9–11]. Adipose and hepatic tissues are the main drivers behind this mechanism and diet-induced severe fatty liver disease could be seen in rodent and human subjects [6, 7, 12–14]. Therefore, advancements in understanding the implications of diet-induced obesity for the whole body are an important factor in health research.

In light of the ongoing debate regarding the interplay between dietary habits, obesity and neurodegenerative diseases, our study aims to shed light on the intricate mechanisms underpinning cognitive decline and Alzheimer´s disease. While numerous studies have established a link between high-energy diets and adverse neurological outcomes including increased oligomeric Amyloid β (Aβ) levels, Aβ plaque and neuroinflammation [15–18], the role of diet-induced obesity in triggering neuroinflammatory processes remains contentious. Notably, dietary components such as fatty acids (FAs) and sugars have increasingly been recognized for their potential to modulate central metabolism and, by extension, the susceptibility to dementia [19–23]. The prevailing hypothesis suggests that metabolic and inflammatory processes initiated by the proliferation of macrophages in adipose tissue and subsequent release of pro-inflammatory cytokines may activate immunomodulatory cascades, leading to neuroinflammation [15, 24, 25]. This perspective is supported by substantial evidence of a rapid and transient microglia activation and proinflammatory cytokine upregulation in the hypothalamus, a critical center for energy homeostasis [26, 27] and hippocampus, implicating neuroinflammation in these regions [15, 28]. However, emerging research challenges this view, documenting cognitive impairments in the absence of significant neuroinflammation in hippocampus and hypothalamus, suggesting that such inflammatory responses may be transient [29, 30]. By integrating these findings, our work aims to elucidate the complexities of diet-induced obesity and its neurological impact, contributing valuable insights to a field marked by divergent evidence.

To date, the molecular mechanisms that connect obesity, glial activity and AD are not fully discovered [31]. Several ways exist to ensure communication between the CNS and periphery, which allow the CNS to adapt and respond to peripheral cues [32, 33]. In neurodegenerative diseases like Parkinson´s disease and AD, emerging evidence indicates that neuroinflammation does not only rely on glial activation, but innate and adaptive immune cells can modulate inflammatory processes in the brain as well [34–36]. In human brain samples and animal models of AD, immunohistochemical experiments revealed substantial involvement of peripheral innate and adaptive immune system components in the pathogenesis. For example in multiple sclerosis mouse models, self-antigen-recognizing T cells have been identified in brains to act as primary drivers of the autoimmune response [37, 38]. Furthermore, infiltration of bone marrow-derived monocytes into CNS was triggered by a high-fat diet [39]. Study results on the impact of peripheral immune cells on AD pathology are however still not in agreement [40–44].

In this study, we show through a multimodal and multiparametric approach that the consumption of a palatable, high-caloric WD during early to mid-life can cause several systemic effects affecting the peripheral and central metabolism. Using the APPPS1 mouse model, a mouse model of early accelerated amyloidosis [45], we identified changes in brain metabolism after disease development using different positron emission tomography (PET) tracers and employed ^1^H- magnetic resonance spectroscopy (^1^H-MRS) and metabolomics to investigate systemic alterations. We first investigated changes in cerebral glucose metabolism, using 2-deoxy-2-[^18^F]fluoro-D-glucose ([^18^F]FDG), a well-established PET marker, widely used to investigate cerebral abnormalities. Second, the diet-induced changes in fatty acid metabolism were analyzed using the long-chain fatty acid surrogate 14(R,S)-[^18^F]fluoro-6-thia-heptadecanoic acid ([^18^F]FTHA). Third, to assess diet-induced neuroinflammatory changes in the brain we used the translocator protein (TSPO) tracer [^18^F]GE-180, which is a surrogate marker for neuroinflammation found mainly on activated glia cells after neuronal damage and inflammation [46–48]. In addition, we performed flow cytometric and metabolic analyses ex vivo to investigate the metaflammation profile of the animals in-depth during WD feeding. With PET, we obtained complementary results showing that diet-induced obesity (DIO) and AD had altered brain glucose and fatty acid metabolism throughout the brain, which are independent of the Aβ pathology and elevated glial reactivity pattern. Moreover, we identified T cells as an additive factor in the interplay of AD pathology and metaflammation and provide further insight into the multifaceted dynamic of glial contribution to neuroinflammation in a model of overnutrition and AD. The imbalance of key plasma metabolites and liver lipids in the periphery, along with the disruption of glucose and fatty acid metabolism in the brain, underscores the importance of a healthy lifestyle and provides further insight into the complex interplay of the brain-liver-fat axis.

## Materials and methods

### Animals

This study was performed in double transgenic APPPS1-21 mice (B6.Cg-Tg(Thy1-APPSw,Thy1-PSEN1*L166P)21Jckr; APPPS1, *n* = 21) that co-expressed the human Swedish double mutation APP KM670/671NL and the L166P mutated human PS1 under the control of neuron-specific Thy-1 promotor. This model shows accelerated amyloid deposition at six weeks of age, accompanied in further age by microglial reactivity to rising pathology [45]. As controls, wild-type C57BL/6J mice (WT, *n* = 23) were used. In transgenic and wild-type groups, male and female mice were investigated. APPPS1 breeding pairs were kindly provided by Prof. Mathias Jucker and bred at the animal facility of the University Hospital Tübingen (Germany). WT and APPPPS1 were housed with litter mates of the same sex, thus housing was genotype mixed with up to five mice per cage. Animals housed in genotype mixed groups in individually ventilated cages with food and water ad libitum in a 12-h light/dark cycle. All procedures, including humane endpoints (set on a combination of factors: (1) occurrence of behavioural changes (e.g. apathy); (2) breathing difficulties or a sharp increase in the respiratory rate; or (3) a weight loss of at least 20% compared to control animals of the same age, sex and diet), were performed in accordance with German federal regulations on the use and care of experimental animals and approved by the local authorities [Regierungspräsidium Tübingen (R06/21 G)].

### Diet and study design

**Fig. 1:**
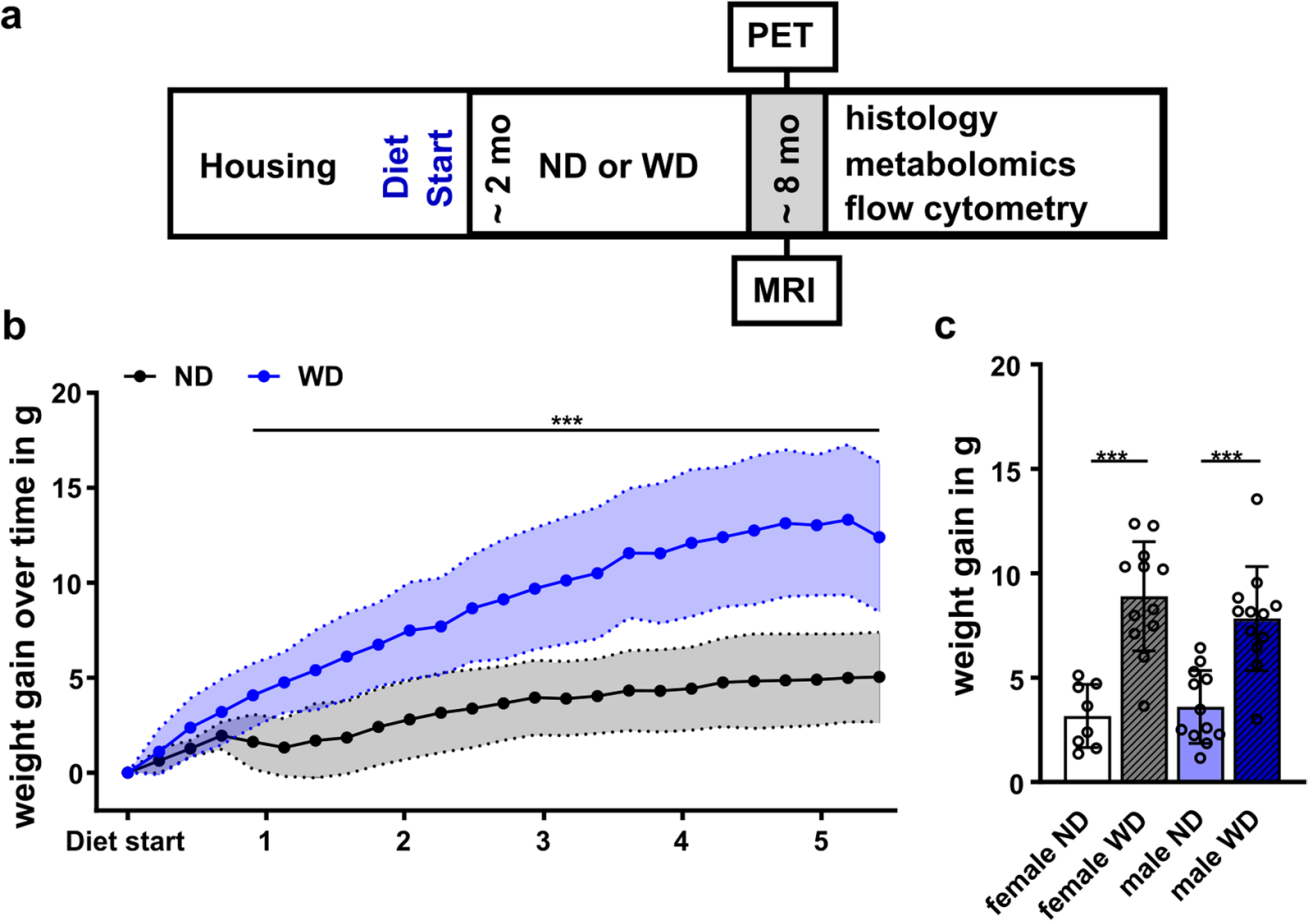
Study design and weight. **a** General study design of in vivo and ex vivo experiments. Western diet (WD) or the normal rodent diet (ND) feeding period started at 2 months of age and continued over 24 weeks. At ~ 8 months, imaging (PET, MRI), flow cytometry, metabolomics, and histology were performed. **b** Mean weight gain ± SD between ND-fed (black) and WD-fed (blue) animals over 24 weeks starting on the day of the diet change. **c** Mean weight gain between females and males fed an ND (blank white, blue) or WD (striped grey, blue). ***p < 0.001. Weight gain over time using student's t-test. Comparison of sex and diet with two-way ANOVA, post hoc Tukey corrected for multiple comparisons. ND, normal rodent diet; WD, western diet; WD (*n* = 24, male = 12, female = 12), ND (*n* = 20, male = 11, female = 9)

Mice were fed a normal rodent diet (V1534-000, ssniff, Soest, Germany) until start of the feeding period. Starting at the age of 2.1 ± 0.1 months, animals were either fed (WD (E15721-347, ssniff, Soest, Germany) or ND (V1534-000, ssniff, Soest, Germany) for a total of 6 months (Fig. 1a). Compared to ND, WD contains higher percentages of fat, sugar, and protein which were accompanied by a shifted balance of fatty acids, minerals and trace elements (Additional file 1: Table S1). To ensure the stability of the dietary constituents, WD was entirely replaced once per week. For ND-fed animals, weight was registered starting at 2.9 ± 0.5 months (*n* = 20; APPPS1 = 9 and WT = 11), whereas for WD-fed animals, registration started at 2.1 ± 0.1 months (*n* = 24, APPPS1 = 12 and WT = 12). The weighting of animals was conducted weekly. At 7.7 ± 0.4 months, the animals underwent in vivo PET and MR imaging over one month and were sacrificed for further flow cytometric, metabolic, and histological analyses (Fig. 1a). Notably in vivo and ex vivo results include individual dropouts of experimental animals due to e.g., technical problems during procedure and/or analyses. Animal numbers per experiment and group are shown in Additional file 1: Table S2. For PET following exclusions were made: for [^18^F]FDG WT-ND group in one animal tracer injection was para; for [^18^F]GE-180 a number of scans had to be excluded due to attenuation correction problems on a scanner (incl. one WT-ND; three WT-WD; two APPPS1-WD). For other missing data either measurement or tracer production failed. Re-measurements were not manageable due to animal welfare and the availability of scanners and tracers. For ex vivo flow cytometry/metabolomics: APPPS1-ND one animal died, WT-WD one animal died, one did not undergo experiments, APPPS1-WS one animal died, and two did not undergo experiments. Only flow cytometry: two WAT probes were contaminated during pre-processing. In all experiments, mice underwent imaging in alternating sequence and ex vivo experiments in mixed groups of wild-types and transgenics as well as sex.

### Radiotracer synthesis

Briefly, using the ^18^O(p,n)^18^F nuclear reaction fluorine-18 was produced as [^18^F]fluoride by proton irradiation of [^18^O]H_2_O (Rotem, Leipzig, Germany) at the Tübingen PETtrace cyclotron (GE Healthcare, Uppsala, Sweden).

[^18^F]FDG was synthesized in a TRACERlab MX_FDG_ synthesizer (GE Healthcare, Liège, Belgium) as described previously, using mannose triflate (ABX, Radeberg, Germany) as a precursor [49]. Quality control was performed according to Ph. Eur. guidelines. Particularly, radiochemical purity, as determined by thin-layer chromatography (TLC), was > 95%. Molar radioactivities were > 50 GBq/µmol at the end of synthesis.

[^18^F]FTHA was synthesized using the method from DeGrado [50] with modifications on a modified TRACERlab FX_F-N_ synthesizer (GE Healthcare, Münster, Germany). Briefly, 2 µL of the precursor benzyl-14-(R,S)-tosyloxy-6-thiaheptadecanoate (ABX, Germany) in 1 mL of acetonitrile were reacted with a mixture of aceotropically dried [^18^F]fluoride, 15 mg of Kryptofix 2.2.2. and 3.5 mg K_2_CO_3_ at 110 °C for 5 min. After hydrolysis with 350 µL of 0.14 N KOH (110 °C, 5 min) 0.3 mL of 6.5% sulfuric acid was added for neutralization. The product was purified using HPLC (Supelcosil ABZ + ; 10 × 250 mm; H_2_O/MeOH 80/20 with 1% H_3_PO_4_; 5 ml/min; detection: UV 216 nm and NaI(Tl)). The product was obtained in uncorrected yields of 15 ± 5% (*n* = 13), corresponding to 9.3 ± 3.3 GBq of isolated [^18^F]FTHA, after irradiations using 35 to 60 µA for 40 to 60 min. Radiochemical purity as determined by TLC was > 90%. Molar activities were > 50 GBq/µmol at the end of synthesis.

[^18^F]GE-180 was synthesized according to Wickstrøm et al. [51] using a FASTlab synthesizer with single-use disposable cassettes (GE Healthcare, Germany) according to the manufacturer’s instructions. Quality control was performed via HPLC, yielding the product in chemical purity above 90% and high molar radioactivity above 600 GBq/µmol at the end of the synthesis.

### PET imaging

PET studies were performed in C57BL/6J and APPPS1 littermates of each group (ND and WD) over 4 weeks. Animals were anesthetized by using isoflurane (carrier gas 100% oxygen at 1 L/min, 5% for induction, 1.2–1.5% maintenance) and body temperature was maintained at 37 °C throughout the studies using mouse beds with temperature feedback control (Medres, Cologne, Germany and Jomatik, Tuebingen, Germany). All PET scans were performed using an Inveon dedicated small-animal microPET scanner (Siemens Healthcare, Knoxville (TN), USA), and scans were acquired dynamically for 60 min, immediately followed by a 14 min [57] Co transmission scan as well as correction of dead time, random and scatter events. Mice were positioned in the center of the field of view and injected intravenously (*i.v.*) into a lateral tail vein with 12.0 ± 0.3 MBq [^18^F]FDG (WT-ND *n* = 10; APPPS1-ND *n* = 7; WT-WD *n* = 7; APPPS1-WD *n* = 8), 14.4 ± 2.3 MBq [^18^F]FTHA (WT-ND *n* = 8; APPPS1-ND *n* = 8; WT-WD *n* = 8; APPPS1-WD *n* = 7) and 13.5 ± 2.5 MBq [^18^F]GE-180 (WT-ND *n* = 8; APPPS1-ND *n* = 7; WT-WD *n* = 9; APPPS1-WD *n* = 10) on consecutive days with at least one day of recovery. Mice were measured with the PET tracers in different order depending on tracer availability. The mice recovered after each scan on a heating pad in an empty cage, and their health was monitored by the researcher.

### PET image reconstruction and data analysis

List-mode data for all scans were histogramed in 23 frames (8 × 30 s, 6 × 60 s, 7 × 300 s, and 2 × 450 s) and reconstructed with a two-dimensional ordered subsets expectation maximization (OSEM2D) algorithm with an image zoom of 2 and a 256 × 256 matrix using Inveon Acquisition Workplace (Siemens Healthcare, USA). Volume-of-interest (VOI) and voxel-wise analyses were performed on reconstructed images using PMOD software v3.2 (PMOD Technologies, Zürich, Switzerland) and statistical parametric mapping SPM 12 (Wellcome Trust Center for Neuroimaging, University College London, United Kingdom). Individual PET images were co-registered to a predefined mouse brain template Mouse-Mirrione-T2 (PMOD technologies), and a whole-brain VOI as well as a brain-region specific atlas were applied [52, 53]. The anterior prefrontal cortex area was removed from the cortex VOI to avoid spill-over effects from the harderian glands. The following brain areas were analyzed: cortex (CTX), hippocampus (HIP), cerebellum (CB), and hypothalamus (HYP). Time activity curves (TACS) were extracted and standardized uptake values (SUVs) for each animal were calculated. For comparison of uptake in all four groups, the mean SUV was evaluated between 30- and 60-min post injection (*p.i.*).

For voxel-wise analysis, PET images were automatically overlaid to the Mouse-Mirrione-T2 atlas as reference (PMOD technologies). Differences between groups for each PET tracer were identified using a general linear model (GLM) available in SPM 12. After estimating GLM, statistical parameter maps were generated by interrogating the outcome using contrast vectors. A one-way ANOVA without post hoc correction was applied. Contrasts were compared between groups using no further masking or determined voxel clusters. The significance threshold was set for the tracers individually. Images were prepared using dedicated software (MRIcron [54]).

### ^*1*^*H- magnetic resonance spectroscopy (*^*1*^*H-MRS) of the liver*

For ^1^H-MRS on a 7 T BioSpec 70/30 MR scanner (Bruker BioSpin GmbH, Ettlingen, Germany) equipped with a gradient insert, animals were anesthetized using isoflurane (carrier gas oxygen 100% at 1 L/min, 5% for induction, 1.2–1.5% maintenance). Animal body temperature was maintained by placing mice on an MR-compatible water-warmed mouse bed (Jomatik, Tuebingen, Germany). During the whole acquisition, breathing was monitored using a specialized MR breathing pad. Mice were positioned in the center of a ^1^H volume coil with an inner diameter of 86 mm. For correct positioning of the liver voxel, an anatomical T2-weighted TurboRARE protocol (TR = 800 ms; TE = 37.63 ms; FOV = 74 × 32 × 18; image size = 296 × 128 × 72) was acquired. Next, B_0_ map (TR = 30 ms; FOV = 60 × 60 × 60 mm^3^, Averages = 1) was acquired. After placing the voxel (3 × 3 × 3 mm^3^), avoiding major hepatic blood vessels, the localized shim was acquired resulting in mean shim values of 52.4 ± 13.3 Hz. For spectral acquisition, a stimulated echo acquisition mode (STEAM; TR = 1500 ms; TE = 3 ms; averages = 512) with and without water suppression (VAPOR) was used. All sequences were acquired using Paravision software v6.0.1 (Bruker, Ettlingen, Germany).

Spectral analysis was performed using LC Model analysis software v6.3-1L (Stephen Provencher, Oakville, ON, Canada) [55]. Subsequently, lipid peaks were evaluated according to Ye et al. 2012 [56], and the following lipids were extracted: Lip09, Lip13, Lip16, Lip21, Lip23, Lip28, Lip41, Lip43, Lip52, and Lip53. Lipids with a standard deviation (SD) > 20 were excluded; thus, animal numbers differ between lipids [57] (Additional file 1: Table S2). Liver fat composition, including lipid mass (LM); fractional lipid mass (fLM), saturated lipid component (SL), fraction of unsaturated lipids (fUL), fraction of saturated lipids (fSL), fraction of polyunsaturated lipid (fPUL), fraction of monounsaturated lipids (fMUL), and mean chain length (MCL) was calculated as described previously [56]. For LM and fLM lipid peaks Lip13 + Lip16 and Lip21 + Lip23 + Lip28 were united to reduce SD below 20 and hence include all animals into the calculation.

### Ex vivo experiments

Following the completion of in vivo measurements, the mice were anesthetized by using isoflurane (carrier gas oxygen), and blood was retro-orbitally taken. Then, the mice were sacrificed through asphyxiation with CO_2_ and perfused through the left ventricle with 20 mL of cold phosphate-buffered saline (PBS), and brain and white adipose tissue (WAT) were removed for ex vivo analysis. Brain tissue was then either paraffin-embedded for histological analysis or further worked-up for cytometric analyses.

### Flow cytometry

Immune cell isolation was performed as described in Hoffmann et al. 2019 [58]. Briefly, brains and WAT of the abdominal cavity were isolated and chopped into small pieces. Tissue was digested for 45 min in 1 mg/mL Collagenase IV (Sigma Aldrich, St. Louis, Missouri, USA) in DMEM supplemented with 5% FCS and 10 mM HEPES at 37 °C. Then, the digested tissue was washed through a 70 µm mesh cell strainer with 1% FCS in PBS. Brain homogenates were centrifuged for 5 min (4 °C, 400 rcf) and resuspended in 70% percoll (in PBS; GE Healthcare, CA, Illinois, USA) layered under 37% percoll solution topped by a 30% percoll solution in a 15 mL Falcon tube and immediately centrifuged 30 min (4 °C, 800 rcf, acceleration of 2 and deceleration of 1). The immune cells, located between percoll layers 70% and 37% after centrifugation, were isolated and centrifuged for 5 min (4 °C, 400 rcf). The remaining erythrocytes were lysed with 3 mL ACK lysing buffer (Lonza, Basel, Switzerland) for 5 min at room temperature. Following washing, the cell suspension was then pipetted into a 5 mL polystyrene tube via a 40 µm cell strainer snap cap (Corning Inc., Corning, New York, USA). Afterward, isolated cells from WAT were counted using cell counting chambers (one-way Neubauer counting chambers, C-Chip, Merck, Darmstadt, Germany). Single-cell suspensions were first stained with viability stain (Zombie NIR fixable viability kit, BioLegend, San Diego, California, USA) followed by either BV510-αCD45 (clone: 30-F11), AF700-αB220 (clone: RA3-6B2), BV605-αCD11b (clone: M1/70), BV711-αLy6G (clone: 1A8), BV785-αCD11c (clone: N418), PE/Cy7-αI-A/I-E (clone: M5/114.15.2) and PE-αF4/80 (clone: BM8) or with PE-αCD45.2 (clone 104), FITC-αCD3 (clone 500A2), AF700-αCD8 (clone 53–6.7), BV521-αCD25 (clone PC61), BV510-αCD44 (clone IM7), PE/Cy7-αCD62L (clone MEL-14), BV650-αCD69 (clone H1.2F3), BV785-αCD127 (clone A7R34), BV711-αPD-1 (clone 29F.1A12). All antibodies were purchased from BioLegend (San Diego, CA, USA). Fc block reagent (CD16/32, clone 93, BioLegend) was included in both antibody panels. The staining took 30 min at 4 °C, and cells were afterward washed three times with PBS (centrifugation 4 °C, 5 min, 400 rcf), fixed in 0.5% formalin, and analyzed on the BD LSRFortessa flow cytometer (BD Biosciences, Franklin Lakes, New Jersey, USA). Analysis was performed with FlowJo software v10.0.7 (BD Biosciences, USA). The gating strategy for both antibody panels is shown in Additional file 1: Fig. S1, S2. Animal numbers for the organs were for brain (WT-ND *n* = 11; APPPS1-ND *n* = 8, WT-WD *n* = 10, APPPS1-WD *n* = 9) and for WAT (WT-ND *n* = 11, APPPS1-ND *n* = 8, WT-WD *n* = 10, APPPS1-WD *n* = 7).

### Metabolomics

For plasma metabolome analysis, blood was collected in an EDTA tube and centrifuged at 4 °C (10 min 2200 rpm) to separate the blood plasma, and aliquots were quenched and snap-frozen with liquid N_2._ A two-phase extraction protocol (polar and lipophilic phases) was applied according to Eggers and Schwudke[59]. In brief: Blood plasma was transferred to 2 mL AFA glass tubes (Covaris Inc, Woburn, Massachusetts, USA) and mixed with ultra-pure water, tert-butyl methyl ether (MTBE, CAS: 1634–04-4, Sigma-Aldrich Chemie, Taufkirchen, Germany) and methanol. Plasma metabolites were extracted using focused ultrasonication (Covaris Inc, USA) applying the following setup: two treatment cycles, 1st: 30 s, Peak Power 125.0, Duty Factor 32.0, Cycles/Burst 400, Avg. Power 40.0. 2nd: 30 s, Peak Power 100.0, Duty Factor 30.0, Cycles/Burst 800, Avg. Power 30.0. Temperature range 5.0 to 15.0 °C. Each cycle repeated five times per sample, the total run time per sample was 5 min. Afterwards, the mixture was centrifuged for 5 min, then the polar (water and methanol) phase was decanted. The resulting solution was evaporated to dryness in three hours with a vacuum concentrator (SpeedVac: Preset 2, Thermo Fischer Scientific Inc., Waltham, Massachusetts, USA). Dried pellets of the polar metabolites were resuspended in deuterated phosphate buffer (75 mM Na_2_HPO_4_, 4% NaN_3_, pH = 7.40) with internal standard 3-(trimethylsilyl) propionic-2,2,3,3-d_4_ acid sodium salt (TSP, CAS: 24,493-21-8). For maximum dissolution, the Eppendorf cups containing solutions were sonicated and then centrifuged for 5 min aiming to remove any solid residue. The supernatant was transferred into 1.7 mm NMR tubes, then centrifuged for 30 s and subsequently placed in a 96-well rack. Samples were kept cooled (6° C) in the NMR automatic sample handling robot unit—SampleJet (Bruker BioSpin, Karlsruhe, Germany) until the measurement. NMR spectra were recorded by a 14.10 Tesla (600 MHz for proton channel) ultra-shielded NMR spectrometer Avance III HD (Bruker BioSpin, Karlsruhe, Germany) with installed 1.7 mm TXI triple resonance microprobe. NMR measurement routine was performed via a 1D CPMG (Carr-Purcell-Meiboom-Gill) experiment to suppress residual background signals from remaining macromolecules like peptides (time domain = 64 k points, sweep width = 20 ppm, 512 scans, 1 h long, temperature 298 K). The recorded free induction decays (FIDs) were Fourier-transformed (FT), and spectra were phase and baseline-corrected.

Bruker TopSpin 3.6.1 software was used for spectra acquisition and processing (offset correction, baseline, and phase correction). ChenomX NMR Suite 8.5 Professional (Chenomx Inc., Edmonton, Canada) was used for metabolite annotation and concentration calculation, additionally, internal ChenomX library was included for a resonance frequency of 600 MHz. The annotated and quantified metabolites of each sample were exported into a spreadsheet file, containing all molar concentrations (before normalization prior statistical analysis) of the full cohort and then further processed with statistical tools.

### Histology and immunohistochemical reactions

Brains were fixed in 4% formalin for 24 h, dehydrated and paraffin-embedded following a standard protocol: 70% Ethanol, 80% Ethanol, 95% Ethanol, 100% Ethanol, Xylene, paraffin wax (58‐60 ºC), and finally, embedded into paraffin blocks. The tissue was then sectioned coronally with a microtome (Microm HS355S Therm Scientific) and the region of interest demarcated using the Mouse Brain Atlas. For each group three randomly chosen specimens underwent histological validation. For histology, 3–5 µm sections were cut and stained with hematoxylin and eosin (H&E). Immunohistochemistry was performed on an automated immunostainer (Ventana Medical Systems, Inc., Oro Valley, Arizona, USA) according to the company’s protocols for open procedures with slight modifications. In short, sections were deparaffinized (Xylene, ethanol 100%–95%–80%) for 8 min and washed. Remaining paraffin was removed by incubation in methanol peroxide (Sigma-Aldrich, St Louis, MO, USA) for ten minutes. The sections were then heated for 20 to 30 min in a 0.01 M citrate-buffered solution and incubated for two hours in Tris buffer (5% FCS). The sections were then incubated with the primary antibodies for two hours and washed 10 times in Tris buffer. Sections were stained with the antibodies CD3 (1:50, Clone SP7, DCS Innovative Diagnostik-Systeme GmbH u. Co. KG, Hamburg, Germany), Iba1 (1:3000, Clone EPR15688. Abcam, Cambridge, UK), beta-amyloid (1:400, Clone Abeta 42, Synaptic Systems, Goettingen, Germany), NeuN XP (1:400, Clone D4G40, Cell Signaling Technology, Danvers, Massachusetts, USA) and GFAP (1:1000, Clone 6F2, Dako, Agilent, Santa Clara, CA, USA). Labelling with the secondary antibody and development was automated using the BasicDAB or iVIEW DAB detection kit. Appropriate positive and negative controls were used to confirm the adequacy of the staining. All samples were scanned with the Ventana DP200 (Roche, Basel, Switzerland) and processed with the Image Viewer MFC Application v.2.3.0. 200 × snapshots were taken in all samples in the cortex, hippocampus, thalamus, choroid plexus, and hypothalamus. The positive cells per field for β-amyloid plaques, CD3, GFAP, and Iba1 positive cells were determined in three snapshots per area of interest. The NeuN quantification was determined for two fields (right and left; 600x) in each of the three cortex cells. NeuN was given as positive cells/total cells. Final image preparation was performed with Adobe Photoshop CS6 (final n number: WT-ND *n* = 2; APPPS1-ND *n* = 3; WT-WD *n* = 3; APPPS1-WD *n* = 3).

### Statistical analyses

In our study, results for weight, ^1^H-MRS lipids, blood glucose, PET tracers, immunological analyses and histologic analyses are presented as mean ± SD. Using Data were analyzed using GraphPad Prism 9.5.1 (GraphPad Software LLC, San Diego, USA) to assess normal distribution and to perform various statistical analyses. To determine statistical significance two-way ANOVA using multiple comparisons with post hoc Holm-Sidak correction (p-value threshold set to α = 0.05) was applied for mean weight gain (sex x diet), metabolomics pyruvate results (genotype x diet), blood glucose (genotype x diet), PET tracers (genotype x diet), immunological analyses (genotype x diet). Pearson´s correlation test was utilized to explore the relationship between cortical uptake of PET tracers (SUV) and immune cell populations (percentage of viable cells).

For the analysis of ^1^H-MRS lipids, multiple unpaired t-tests with post hoc multiple comparison corrections using Holm-Sidak method (p-value threshold set to α = 0.05) were conducted. SPM12 was used to apply a one-way ANOVA without post hoc correction for voxel-wise data analysis.

For metabolomics, MetaboAnalyst 5.0 web server (R-based online analysis tool, www.metaboanalyst.ca) was used for metabolite statistical analysis [60]. Additional checks were performed via MetaboAnalyst 6.0 web server (www.metaboanalyst.ca). To do so, the full cohort spreadsheet with all raw metabolite concentrations was uploaded to the web server. Missing values of metabolites that were below the SNR in one or the other samples were replaced by a small value (20% of the minimum positive value in the original data). Data was further normalized using probabilistic quotient normalization (PQN) [61] in order to account for dilution effects. Next, Pareto scaling was applied for variance stabilization (mean-centered and divided by the square root of the standard deviation of each variable). The data was then analyzed using statistical approaches: one-way ANOVA (analysis of variance), partial least squares discriminant analysis (PLS-DA) and t-testing. Box plot graphical design (and a two-way ANOVA run) was performed in GraphPad Prism 9.5.1 (GraphPad Software LLC).

Results were considered statistically significant with a p value < 0.05.

## Results

### Body weight

To assess the effect of long-term consumption of a WD, WT and APPPS1, animals were fed either ND or a WD starting at the age of 2 months over 24 weeks (Fig. 1a). In vivo imaging took place over four weeks and was complemented via flow cytometry, histology and metabolomic experiments (Fig. 1a). Weight monitoring showed a significantly faster weight gain in WD-fed mice compared to ND-fed animals over time (Fig. 1b) with a 2.5-fold higher mean weight gain in the WD group (start to end: 8.7 ± 3.8 g) compared to the ND group (start to end: 3.3 ± 1.4 g). Mean weight gain between males and females did not differ but confirmed dietary effects (Fig. 1c, Df = 1, F = 54.7, p_ANOVA_ < 0.001).

### Liver fat composition and metabolomics

**Fig. 2:**
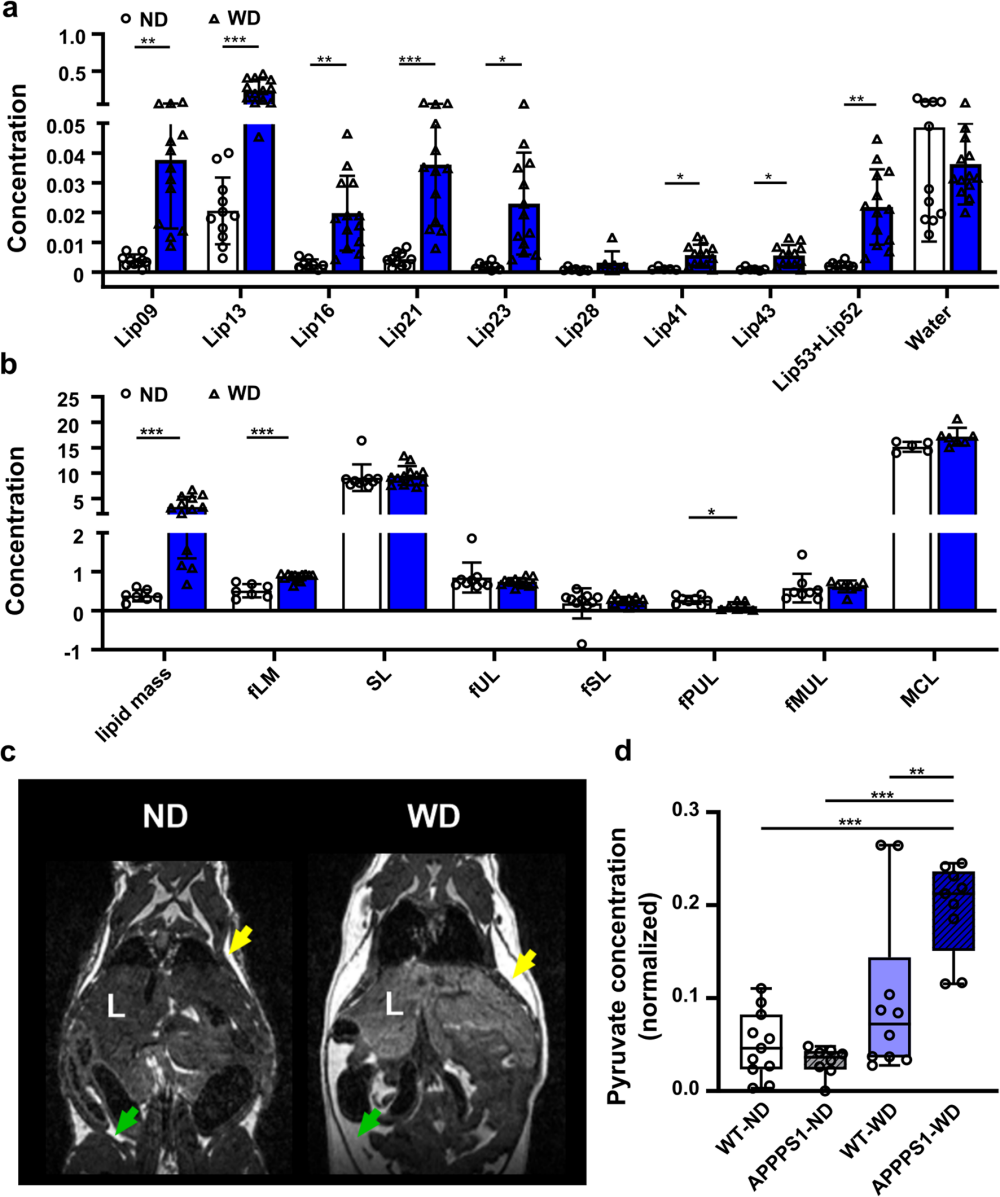
MR-based lipid analysis and metabolomics. ^1^H MRS of hepatic lipid composition and metabolomic results between ND and WD-fed animals. **a** single lipids are depicted according to their chemical shift, indicating changes between ND (white, circles)- and WD-fed animals (blue, triangles). **b** Calculated lipid compositions using the single lipid peaks. **c** Exemplary contrast-normalized T2-weighted images illustrating fat depots in ND- and WD-fed mice. Subcutaneous fat is marked with yellow arrows; abdominal fat is marked with green arrows. **d** Box plot of pyruvate changes between the four mice groups. where the following components are defined: center line, median; box limits, 25–75 percentiles; whiskers, minimum to maximum; and all points are shown. WT-ND *n* = 11 (male = 7, female = 4), APPPS1-ND *n* = 8 (male = 4, female = 4), WT-WD *n* = 10 (male = 4, female = 6), APPPS1-WD *n* = 9 (male = 4, female = 5). ^1^H MRS results were analyzed using multiple unpaired t-tests with post hoc multiple comparison corrections using the Holm-Sidak method (*p*-value threshold set to α = 0.05). Metabolomics with two-way ANOVA, post hoc Holm-Sidak correction for multiple comparisons. fLM, fractional lipid mass; SL, saturated lipid component; fUL,  fraction of unsaturated lipids; fSL,  fraction of saturated lipids; fPUL,  fraction of polyunsaturated lipids; fMUL,  fraction of monounsaturated lipids; L,  liver; Statistical significance: *p < 0.05, **p < 0.01, ***p < 0.001

By using ^1^H- magnetic resonance spectroscopy (^1^H-MRS), we next aimed to assess liver fat composition non-invasively by spectral analysis (Additional file 1: Fig. S3). ^1^H-MRS analyses revealed higher lipid fractions with different chain lengths in WD-fed animals (Fig. 2b, absolute values presented in Additional file 1: Table S3). We observed a tenfold higher calculated lipid mass (ND: 0.36 ± 0.15; WD: 3.35 ± 2.00) and fractional lipid mass (ND: 0.48 ± 0.19; WD: 0.88 ± 0.09) in WD-treated mice (Fig. 2c, Additional file 1: Table S3). Interestingly, among the unsaturated lipid components, only the calculated fraction of polyunsaturated lipids (fPUL) was significantly smaller in WD livers (ND: 0.29 ± 0.10; WD: 0.12 ± 0.10), whereas the saturated lipids (SL and fSL) did not differ between both diets (Additional file 1: Table S3). Thus, WD leads to a higher accumulation of hepatic lipids. Overall, MR images indicated higher body fat accumulation including abdominal and subcutaneous fat (Fig. 2d).

Plasma metabolite profiles were examined by NMR metabolomics. In total, 24 serum (timepoint 8-months) metabolites from various metabolite classes, such as amino acids, ketone body—3-hydroxybutyrate (3-HB), energy metabolites, and short-chain fatty acids were identified (all metabolomics NMR based summary is reported in the Additional file 1: Table S4; with fitting signals characteristics; chemical shifts (ppm), and normalized (concentrations) averages with corresponding SDs (standard deviations)). We observed a substantial change for pyruvate in APPPS1-WD animals (Fig. 2d; VIP score = 1.2), with a strong diet effect (Df = 1, F = 34.2, p_ANOVA_ < 0.001), small genotype effect (Df = 1, F = 4.57, p_ANOVA_ = 0.04) and interaction between the two factors (Df = 1, F = 9.70, p_ANOVA_ = 0.004). All groups, aside from the ND-WT group, developed significantly higher amounts of 3-HB and isoleucine (Additional file 1: Table S5 group comparisons A–C). Further, in the PLS-DA analysis and t-test, histidine was drastically lowered in the plasma of the APPPS1-WD group (Additional file 1: Table S5 group comparison C, VIP score = 0.7). Moreover, considerable changes were identified for glucose (Additional file 1: Table S5 group comparison B, VIP score = 4.6). Consistently with the higher accumulation of liver fat, animals showed a peripheral misbalance caused by overnutrition. Regression model analysis identified more changes within transgenic and wild-type mice group comparison (Additional file 1: Table S5 groups comparisons A, C). Here, we found lower levels of citrate and succinate—important TCA cycle metabolites, the amino acids phenylalanine and tyrosine, and creatine in transgenic animals.

### Cerebral glucose metabolism

**Fig. 3:**
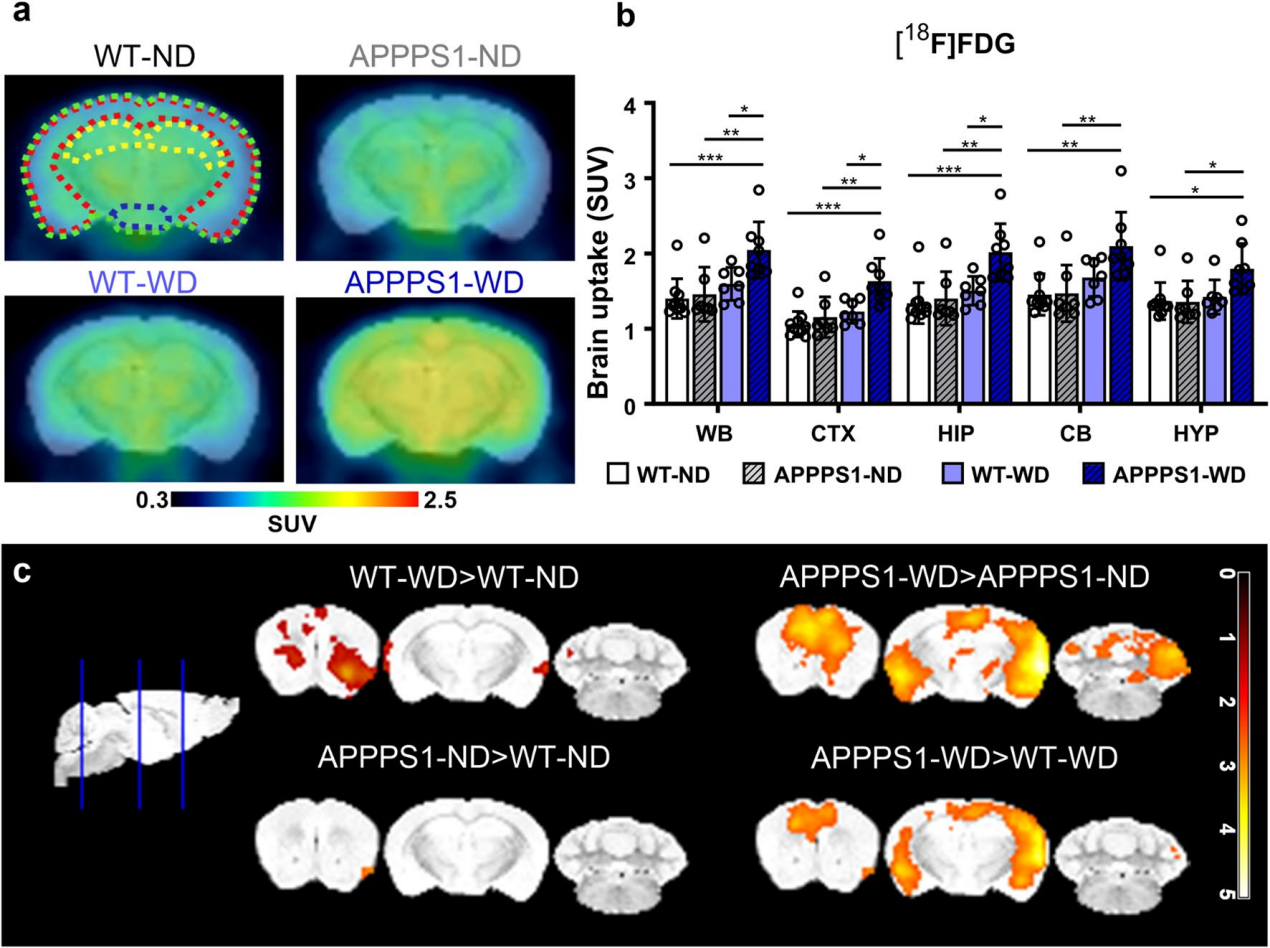
[^18^F]FDG-PET imaging. **a** Comparison of axial brain images of [^18^F]FDG uptake in all four groups indicates a higher uptake in APPPS1-WD mice. Regions are indicated as follows: green = WB; red = CTX; yellow = HIP; blue = HYP. **b** mean SUV (30–60 min *p.i.*) in WB, CTX, HIP, CB, and HYP between all groups. **c** T-maps comparing SUVs are shown with threshold p < 0.01. Individual data points are shown and displayed with mean and SD. WT-ND *n* = 10 (male = 6, female = 4), APPPS1-ND *n* = 7 (male = 3, female = 4), WT-WD *n* = 7 (male = 4, female = 3), APPPS1-WD *n* = 8 (male = 5, female = 3). *p < 0.05, **p < 0.01, ***p < 0.001, post hoc Holm-Sidak corrected for multiple comparisons; WB, whole brain; CTX, cortex; HIP, hippocampus; CB, cerebellum; HYP, hypothalamus

To examine changes in cerebral glucose metabolism, we used [^18^F]FDG-PET. Mean blood glucose values were measured before the imaging and did not differ between groups (Additional file 1: Fig. S4). Representative images of axially positioned brains of each group showed higher [^18^F]FDG uptake in the APPPS1-WD group. In contrast, no changes between the other groups were observed visually (Fig. 3a). Mean SUV of [^18^F]FDG in the whole brain displayed an effect between genotypes and the diets (Table 1a) with pronounced values in WD-fed APPPS1 mice compared to the other conditions (Fig. 3b). No significant interaction in [^18^F]FDG SUV between the other groups could be seen. Further region-based quantification highlighted similar effects for the cortex and hippocampus (Table 1a) with differences between the APPPS1-WD group and the other groups, underlining an overall brain effect. Other analyzed regions showed diet effects only (Table 1a). Voxel-wise analysis confirmed a whole-brain effect in the APPPS1-WD group (Fig. 3c). A minor difference in [^18^F]FDG accumulation between WT-ND and WT-WD could be seen in anterior areas, which could not be identified in the prior quantification procedure, indicating a minor effect of WD on [^18^F]FDG in the anterior region of healthy brains. Together with the VOI-based results, [^18^F]FDG showed high uptake in the WD-fed amyloid mouse model.

**Table 1:** Two-way ANOVA results representing degree of freedom (Df), F-value (F), and *p*-value for PET results divided into factors ‘genotype’ and ‘diet’

		Df	F	*p*-value	Df	F	*p*-value	Df	F	*p*-value
		a [^18^F]FDG			b [^18^F]FTHA			C [^18^F]GE-180		
WB	Genotype	1	5.289	0.029*	1	0.453	0.507	1	25.420	< 0.001***
	Diet	1	12.650	0.001**	1	28.850	< 0.001***	1	3.510	0.071
CTX	Genotype	1	9.291	0.005**	1	1.383	0.250	1	47.440	< 0.001***
	Diet	1	15.630	< 0.001***	1	23.040	< 0.001***	1	5.237	0.029*
HIP	Genotype	1	6.770	0.015*	1	0.657	0.425	1	33.150	< 0.001***
	Diet	1	12.360	0.002**	1	25.790	< 0.001***	1	3.942	0.056
CB	Genotype	1	3.042	0.092	1	1.441	0.240	1	5.541	0.025*
	Diet	1	11.870	0.002**	1	27.610	< 0.001***	1	3.013	0.093
HYP	Genotype	1	3.410	0.075	1	0.086	0.772	1	3.486	0.072
	Diet	1	6.365	0.018*	1	25.970	< 0.001***	1	13.300	< 0.001***

Results are shown for a [^18^F]FDG, b [^18^F]FTHA, and c [^18^F]GE-180

WB, whole brain; CTX, cortex; HIP, hippocampus; CB, cerebellum; HYP, hypothalamus

*p < 0.05, **p < 0.01, ***p < 0.001

### Cerebral fatty acid metabolism

**Fig. 4:**
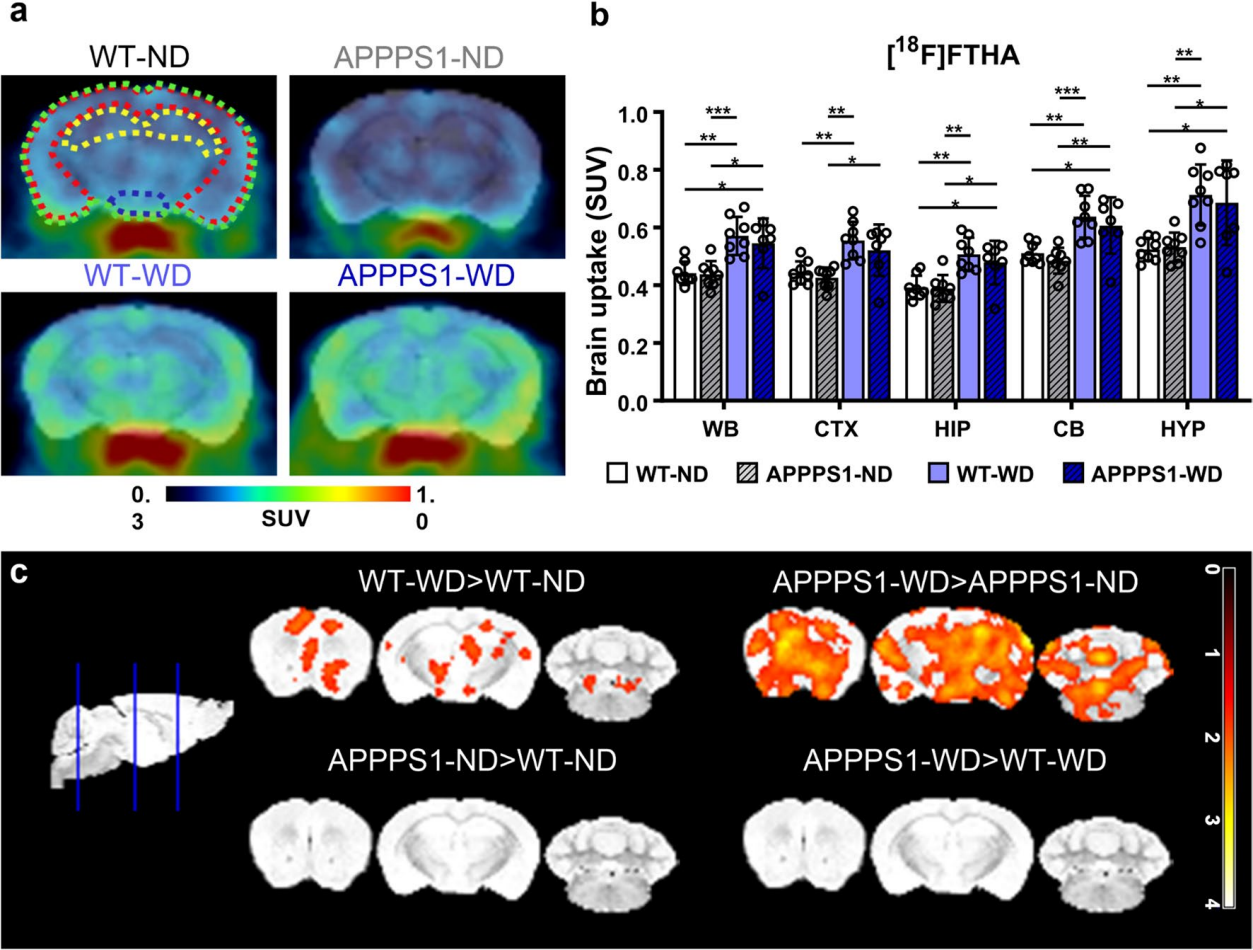
[^18^F]FTHA-PET imaging. **a** Exemplary axial brain images of [^18^F]FTHA uptake display higher uptake in WD-fed mice irrespective of genotype. Regions are indicated as follows: green = WB; red = CTX; yellow = HIP; blue = HYP. **b** Mean SUVs (30–60 min *p.i.*) in WB, CTX, HIP, CB, and HYP for [^18^F]FTHA. **c** Comparison of voxel-wise analysis. The threshold was set to p < 0.05. Individual data points are shown and displayed with mean and SD. WT-ND *n* = 8 (male = 5, female = 3), APPPS1-ND *n* = 8 (male = 4, female = 4), WT-WD *n* = 8 (male = 5, female = 3), APPPS1-WD *n* = 7 (male = 4, female = 3). *p < 0.05, **p < 0.01, ***p < 0.001, post hoc Holm-Sidak corrected for multiple comparisons; WB, whole brain; CTX, cortex; HIP, hippocampus; CB, cerebellum; HYP , hypothalamus

Next, we investigated the impact of WD on fatty acid metabolism in vivo using the long-chain fatty acid analog [^18^F]FTHA. Axial brain images representing [^18^F]FTHA uptake in all groups displayed a higher brain uptake in WD-fed groups (Fig. 4a). VOI-based whole-brain mean SUVs confirmed a significantly higher [^18^F]FTHA uptake in WD-fed animals, both in WT and APPPS1 animals with no differences between genotypes (Fig. 4b, Table 1b). Segmentation of the brain regions could not highlight any region-specific statistical difference in [^18^F]FTHA accumulation, suggesting a whole-brain effect. Overall, the higher uptake of [^18^F]FTHA in WD-fed mice was independent of the genotype in all observed brain regions. Further data analysis on voxel level showed higher signals in WD-fed animals over ND-fed animals in all observed regions in the brain but did not support the identification of more prominent areas (Fig. 4c).

### Neuroinflammation

**Fig. 5:**
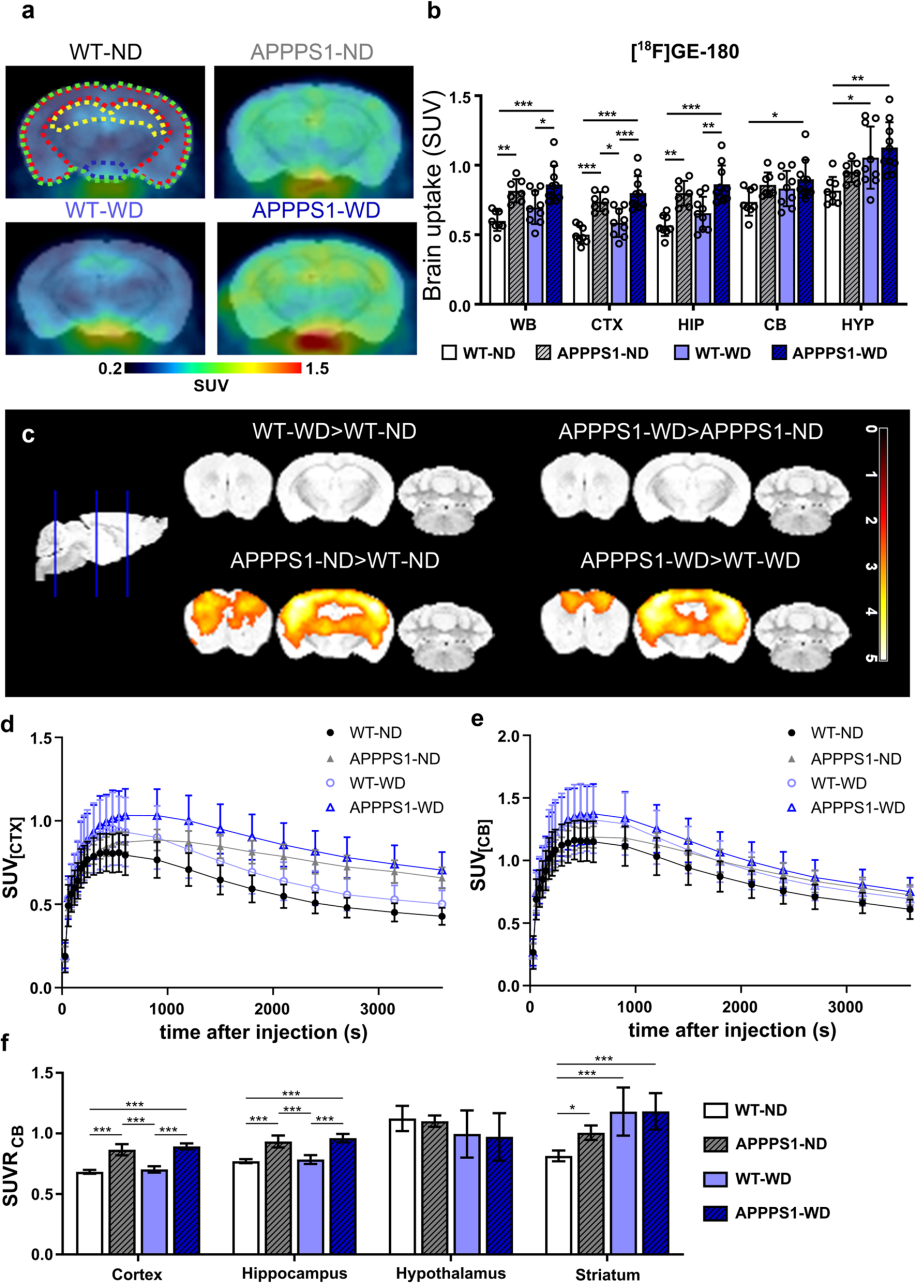
[^18^F]GE-180-PET imaging. **a** Higher uptake of [^18^F]GE-180 in APPPS1 mice compared to WT shown in representative axial brain images. Colored outlines illustrate the analyzed brain regions green = WB; red = CTX; yellow = HIP; blue = HYP. **b** Mean SUVs (30–60 min *p.i.*) in WB, CTX, HIP, CB, and HYP for [^18^F]GE-180 in all groups. **c** Representative images of voxel-wise analyzed SUVs are shown with threshold p < 0.01. Individual data points are shown and displayed with mean and SD. WT-ND *n* = 8 (male = 5, female = 3), APPPS1-ND *n* = 7 (male = 3, female = 4), WT-WD *n* = 9 (male = 5, female = 4), APPPS1-WD *n* = 10 (male = 5, female = 5). *p < 0.05, **p < 0.01, ***p < 0.001; post hoc Holm-Sidak corrected for multiple comparisons; WB,   whole brain; CTX, cortex; HIP, hippocampus; CB, cerebellum; HYP, hypothalamus

Next, we aimed to investigate the influence of WD on neuroglial reactivity in WT and APPPS1 mice using the TSPO-PET tracer [^18^F]GE-180. Whole-brain SUV analysis showed that [^18^F]GE-180 accumulation was significantly elevated in APPPS1 brains, with small effects of the diet (Fig. 5a, Table 1c), pointing to a genotype-dependent effect. Further region-specific analysis pointed at significant radiotracer accumulation differences in the CTX and HIP, regions typically the most affected by amyloidosis (Fig. 5b, Table 1c). Regions with less to no amyloid burden, however, showed small genotype effects and none to very high diet effects (Table 1c). Tracer uptake over time revealed a higher injection peak in transgenic animals compared to wild-type animals for cortex, whereas for cerebellum peaks did not differ (Fig. 5d, e). Ratios between cortex and cerebellum as well as hippocampus and cerebellum showed significantly higher values in APPPS1 brains than in WT (Fig. 5f). However, no differences between diets were observed. Further voxel-wise comparison highlighted the main [^18^F]GE-180 accumulation differences between WT and APPPS1 brains to be located in CTX and HIP primarily (Fig. 5c). Pearsons’s correlation have shown that there is no correlation between the whole brain tracer uptake except for [^18^F]FDG and [^18^F]FTHA in APPPS1-ND group.

### Immune cell presence in the brain

**Fig. 6:**
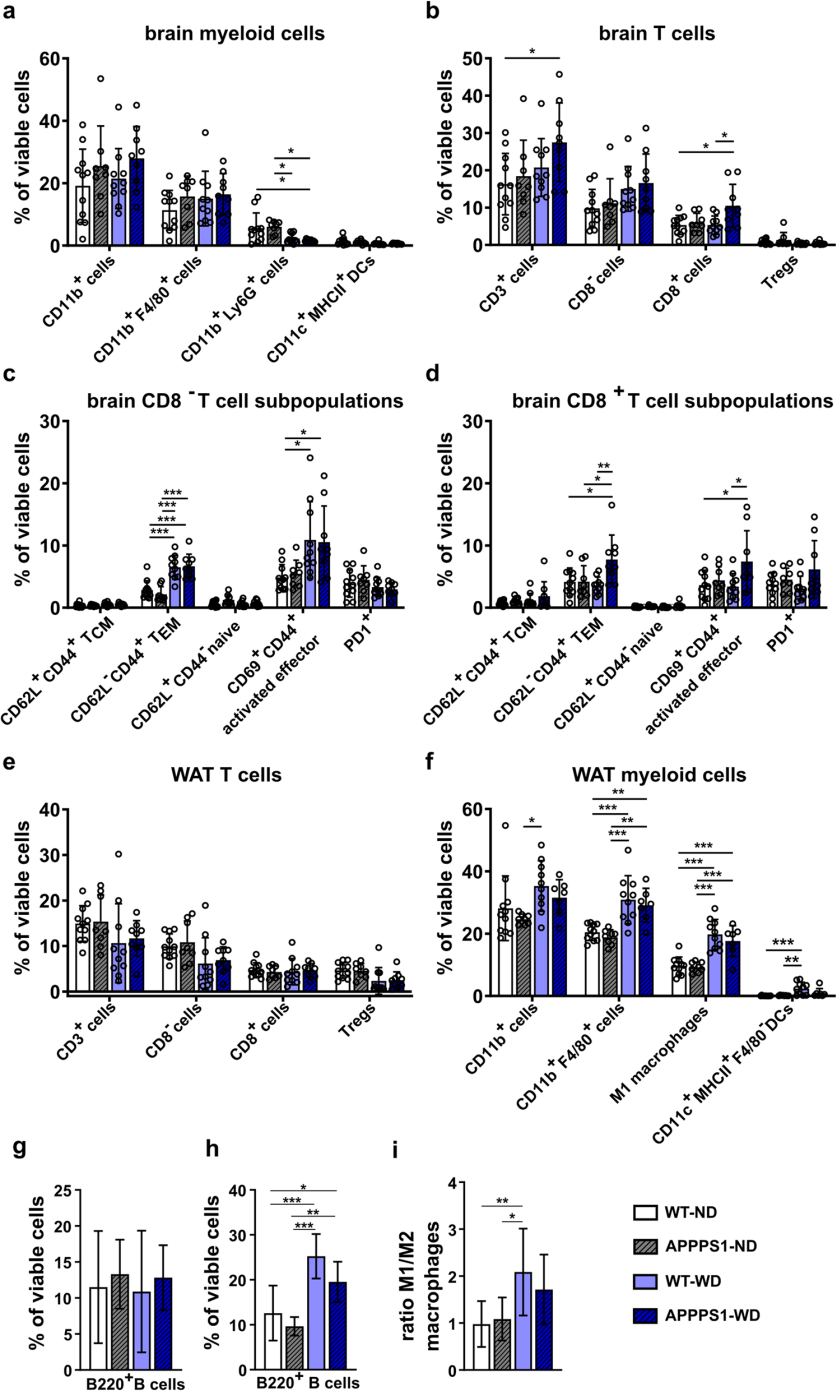
Immune cell analysis of brain and WAT. Brain and WAT immune cell population displayed as the % of viable cells. **a** Brain myeloid cells show only minor changes. **b** CD3^+^ T cells and CD8^+^ T cells are significantly higher in APPPS1-WD mice compared to non-treated WT animals. **c** CD8^−^ T cell subpopulations reveal higher effector memory T cells (T_EM_) and higher activated effector T cells in WD groups. **d** CD8^+^ T cell populations show higher effector memory and activated effector T cell phenotype, but only in APPPS-WD animals. **e** WAT myeloid cell population displays significantly higher CD11b^+^F4/80^+^ macrophages, inflammatory M1 macrophages (CD11b^+^F4/80^+^CD11c^+^), and CD11c^+^MHCII^+^ DCs in WD-fed groups. **f** T cell populations in WAT reveal no differences between groups. **g** Flow cytometry results show no changed B cell populations in the brain, but **h** significantly elevated in WAT of WD-fed mice. M1/M2 ratio is higher in WD-WAT (**i**). Results in mean ± SD; *p < 0.05, **p < 0.01, ***p < 0.001, post hoc Holm-Sidak corrected for multiple comparisons; Brain (**a**-**d**; **g**): WT-ND *n* = 11 (male = 7, female = 4), APPPS1-ND *n* = 8 (male = 4, female = 4), WT-WD *n* = 10 (male = 6, female = 4), APPPS1-WD *n* = 9 (male = 5, female = 4). WAT (**e**; **f**; **i**): WT-ND *n* = 11 (male = 7, female = 4), APPPS1-ND *n* = 8 (male = 4, female = 4), WT-WD *n* = 10 (male = 6, female = 4), APPPS1-WD *n* = 7 (male = 3, female = 4). *p < 0.05, **p < 0.01, ***p < 0.001. DC,  dendritic cells; T_CM_,  central memory T cells; T_EM_ = effector memory T cells; Tregs = regulatory T cells

In order to uncover the potential immune changes underlying the brain metabolism and to go beyond glial activation as a marker for brain inflammation as seen by in vivo PET, we investigated changes in immune cell infiltration in mice brains. Leukocytes were extracted from brains and sorted between innate and adaptive immune cells by two individual antibody panels, making it possible to check for subtypes. Brains of the APPPS1-WD group had less CD11b^+^Ly6G^+^ neutrophils (Fig. 6a; WT-ND vs. APPPSS1-WD p = 0.02; APPPS1-ND vs. APPPS1-WD p = 0.02) than ND brains showing a strong diet effect in this group (Table 2a). No differences could be detected for CD11b^+^ myeloid cells, CD11b^+^F4/80^+^ macrophages, and dendritic cells (CD11c^+^MHCII^+^ DCs) between the groups, however, ANOVA revealed a diet effect in DCs (Table 2a). Similar results were observed for the WT-WD group (CD11b^+^Ly6G^+^ neutrophils: APPPS1-ND vs. WT-WD p = 0.05). When investigating T cell infiltration (Fig. 6b), the APPPS1-WD group showed a tendency of increased number of CD3^+^ cells compared to WT-ND controls (p = 0.0524) and showed a diet effect (Table 2b). Further discrimination between CD8^+^ cytotoxic T cells and CD8^−^ T cells revealed elevated CD8^+^ T cells in APPPS1-WD brains compared to WT groups (APPPS1-WD vs. WT-ND p = 0.01; vs. WT-WD p = 0.02). No difference within the groups was observed for CD25^+^CD127^−^ regulatory T cells (Tregs), but tendencies towards diet involvement (Table 2b). B cell populations did not differ either (Fig. 6g). To determine the T cells’ possible function in the brain, we next investigated the T cell subtypes. Here, WD-fed animals had a higher CD8^−^ T cell effector memory (T_EM_) phenotype, whereas central memory (T_CM_) and naive T cell populations did not change (Fig. 6c, Table 2c). Moreover, these groups had a higher proportion of CD69^+^ lymphocytes, indicating activation, compared to WT-ND controls (Table 2c). In comparison, CD8^+^ T cell subpopulations were elevated only in the APPPS1-WD group (Fig. 6d). Here, a higher percentage of effector memory T cells (T_EM_) compared to the other groups was detected (Fig. 6d; WT-ND versus APPPS1-WD; p = 0.02; APPPS1-ND versus APPPS1-WD p = 0.04; WT-WD versus APPPS1-WD p = 0.009) and a trend towards higher CD69^+^CD44^+^ activated effector population compared to the WT groups emerged (Fig. 6d, WT-ND versus APPPS1-WD p = 0.05; WT-WD versus APPPS1-WD p = 0.04). The immune checkpoint PD1^+^ revealed no differences in CD8^−^ profile (Fig. 6c, Table 2c), but a genotype effect in CD8^+^ profile (Fig. 6d, Table 2d). While we detected no pronounced infiltration of innate immune cells, these results indicate that WD initiated T cell involvement which displayed an effector state. Correlations between PET tracers and significant immune cell populations in brain revealed distinct positive association between and CD8-TEMs and [^18^F]FDG cortices (R^2^ = 0.611; *p* = 0.007) and [^18^F]GE-180 cortices (R^2^ = 0.611; *p* = 0.026), but no overall correlation pattern between tracers and other immune cell populations (Additional file 1: Table S6).

**Table 2:** Two-way ANOVA results showing degree of freedom (Df), F value (F), and significance (*p*-value) for the flow cytometry data divided into factors ‘genotype’ and ‘diet’

	Factor	Df	F	*p-*value		factor	Df	F	*p*-value
*a*	*Brain myeloid cells*				*b*	*Brain T cells*			
CD11b^+^cells	Genotype	1	3.095	0.088	CD3^+^cells	Genotype	1	2.304	0.138
	Diet	1	0.435	0.514		Diet	1	5.279	0.028*
CD1b^+^F4/80^+^cells	Genotype	1	1.526	0.225	CD8^−^cells	Genotype	1	0.478	0.494
	Diet	1	0.882	0.354		Diet	1	6.554	0.015*
CD11b^+^Ly6G^+^cells	Genotype	1	0.016	0.898	CD8^+^cells	Genotype	1	6.360	0.017*
	Diet	1	16.710	< 0.001***		Diet	1	3.735	0.062#
CD11c^+^MHCII^+^DCs	Genotype	1	0.355	0.555	Tregs	Genotype	1	0.915	0.346
	DIET	1	5.853	0.021*		Diet	1	3.640	0.065#
*c*	*Brain CD8*^*−*^* T cell*				*d*	*Brain CD8*^*+*^* T cell*			
CD62L^+^CD44^+^T_CM_	Genotype	1	0.025	0.876	CD62L^+^CD44^+^T_CM_	Genotype	1	1.569	0.219
	Diet	1	5.279	0.028*		Diet	1	1.647	0.208
CD62L^−^CD44^+^T_EM_	Genotype	1	0.225	0.639	CD62L^−^CD44^+^T_EM_	Genotype	1	5.910	0.021*
	Diet	1	55.500	< 0.001***		Diet	1	3.071	0.089
CD62L^+^CD44^−^naive	Genotype	1	4.987	0.032*	CD62L^+^CD44^−^naive	Genotype	1	4.387	0.044*
	Diet	1	2.337	0.136		Diet	1	0.079	0.781
CD69^+^CD44^+^effector	Genotype	1	0.007	0.935	CD69^+^CD44^+^effector	Genotype	1	5.622	0.024*
	Diet	1	14.400	< 0.001***		Diet	1	1.922	0.175
PD1^+^	Genotype	1	0.036	0.851	PD1^+^	Genotype	1	4.426	0.043*
	Diet	1	2.939	0.096		Diet	1	0.320	0.575
*e*	*WAT T cells*				*f*	*WAT myeloid cells*			
CD3^+^cells	Genotype	1	0.067	0.798	CD11b^+^cells	Genotype	1	1.944	0.173
	Diet	1	4.090	0.052#		diet	1	7.339	0.011*
CD8^−^cells	Genotype	1	0.136	0.715	CD11b^+^F4/80^+^cells	Genotype	1	1.003	0.324
	Diet	1	8.452	0.007**		Diet	1	36.400	< 0.001***
CD8^+^cells	Genotype	1	0.005	0.944	M1 macrophages	Genotype	1	0.993	0.326
	Diet	1	0.031	0.861		Diet	1	52.810	< 0.001***
Tregs	Genotype	1	0.00	0.990	CD11c^+^MHCII^+^F4/80^−^DCs	Genotype	1	2.505	0.123
	Diet	1	9.480	0.004**		Diet	1	13.370	< 0.001***

The results are presented: (a) brain myeloid cells, (b) brain T cells, (c) brain CD8- T cell subpopulations, (d) brain CD8 + T cell subpopulations, (e) WAT T cells and (f) WAT myeloid cells

DC, dendritic cells; T_CM_, central memory T cells; T_EM_, effector memory T cells; Tregs, regulatory T cells

*p < 0.05, **p < 0.01, ***p < 0.001

### Immune cell population in white adipose tissue (WAT)

One major hallmark of obesity-induced inflammation is the accumulation and activation of macrophages in adipose tissue [12]. Therefore, we next investigated changes in T cells and myeloid cells in WAT by flow cytometry (Fig. 6e and f). T cell populations in WAT between groups were not different, but showed a diet effect in CD8^−^ populations and Tregs (Fig. 6e, Table 2e). Additionally, B cell populations were significantly higher in WAT of obese compared to lean animals irrespective of their genotype (Fig. 6h).A substantial elevation of macrophage marker F4/80^+^ was detected in both WD-fed groups (Fig. 6f). Further examination of the pro-inflammatory macrophage M1 phenotype using CD11c^+^ [62] displayed higher populations in WD groups. The ratio of M1 to M2 F4/80^+^ macrophages was shifted towards a higher M1 portion in WD-fed animals (Fig. 6i). To ensure that we see dendritic cells (DCs) and not M1 macrophages as all APCs express MHCII and CD11c in WAT, the DC population was additionally gated negative for F4/80. DC populations were elevated in WD groups, in which WT-WD showed the greatest differences (Fig. 6f). Interestingly, all examined populations had a strong diet effect in ANOVA analyses (Table 2f).

### Immunohistochemistry (IHC)

**Fig. 7:**
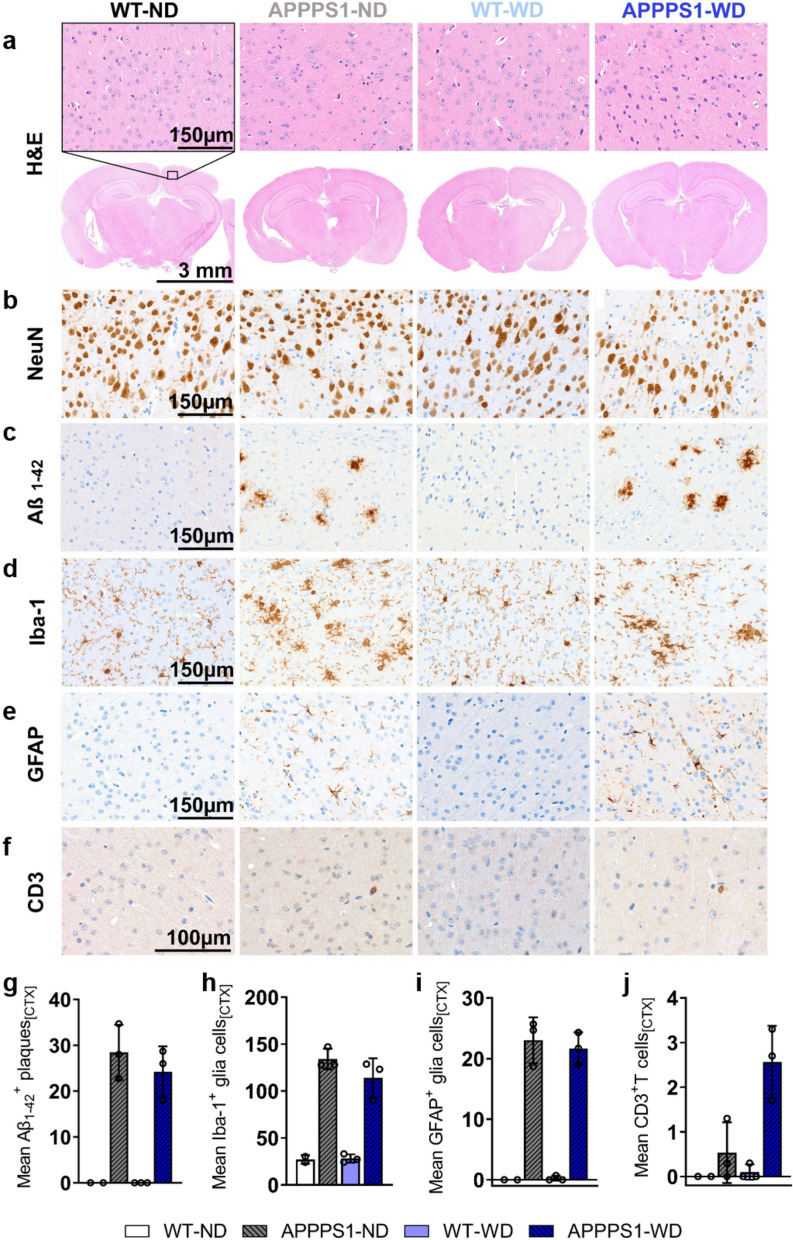
Histological analysis—cortex. Histological staining results are depicted in representative images per group per staining staining for cortices of all groups. **a** H&E staining between groups as overview (Scale bar 3 mm) and magnification (Scale bar 150 µm). The black rectangular depicts magnification area. **b** NeuN staining reveals no differences between groups. **c** Amyloid plaques stained specifically with Aβ_1-42_ antibody were visible in APPPS1 animals (Scale bar 150 µm). **d** Microglia staining using Iba-1 as a marker shows a ramified/resting phenotype in WT brains, whereas activated amoeboid phenotype of microglia in transgenic AD animals was observed (Scale bar 150 µm). **e** GFAP staining shows reactive astrocytes in APPPS1 animals in CTX. No GFAP + cells were found in WT animals (Scale bar 150 µm). **f** CD3^+^ staining revealed T cells in APPPS1 cortices (Scale bar 100 µm). **g** Mean ± SD Aβ_1-42_ positive plaques in cortices show plaque load in transgenic mice but no difference between diets. **h** Mean ± SD Iba-1^+^ cells are elevated in APPPS1 cortices independent of the diet. **i** No mean ± SD GFAP^+^ cells in WT mice, but elevated diet-independently in APPPS1 mice. **j** Higher mean ± SD CD3 positive T cells in APPPS1-WD group compared to the other groups. CTX = cortex; HIP = hippocampus; HYP = hypothalamus. WT-ND *n* = 2; APPPS1-ND *n* = 3; WT-WD *n* = 3; APPPS1-WD *n* = 3

**Fig. 8:**
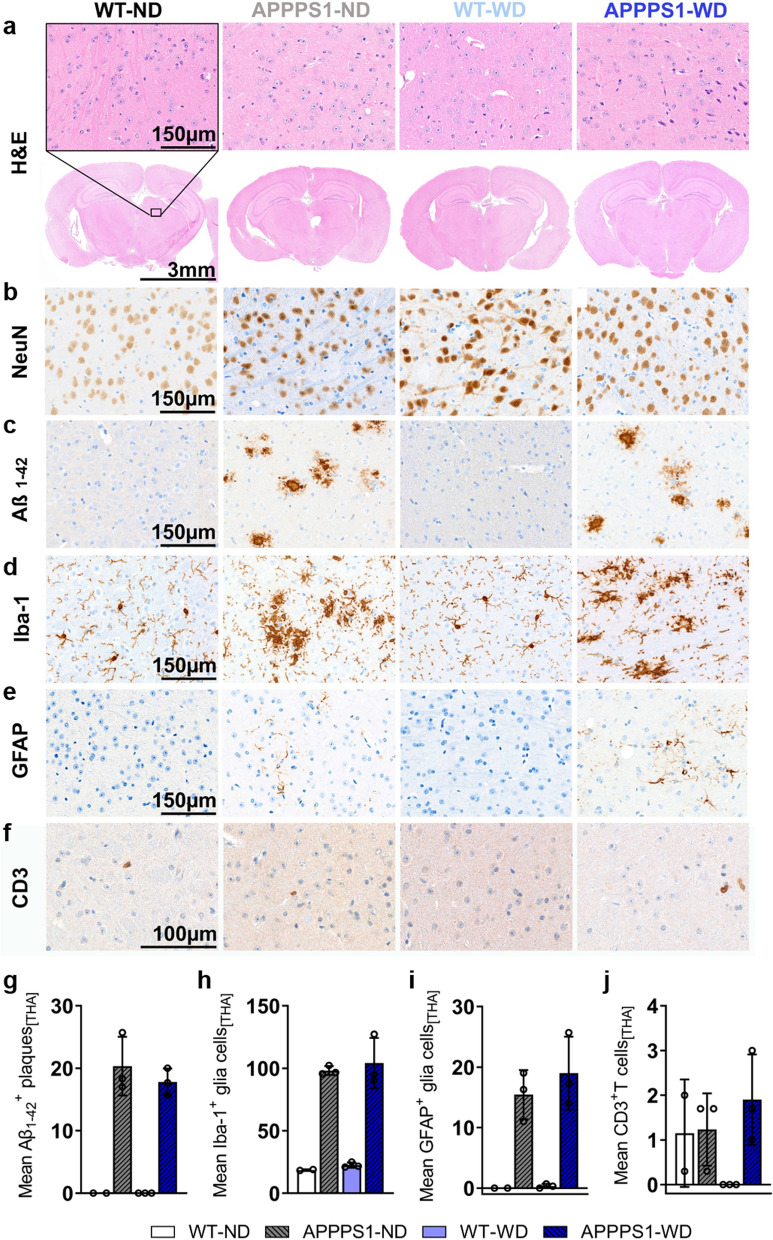
Histological analysis—Thalamus. Histological staining results are depicted in representative images per group per staining for the thalamus (THA) in all groups. **a** H&E staining between groups as overview (Scale bar 3 mm) and magnification (Scale bar 150 µm). The black rectangular depicts magnification area. **b** No difference in NeuN^+^ cells between groups. **c** Aβ_1-42_ positive plaques were visible in APPPS1 animals (Scale bar 150 µm). **d** Microglia staining using Iba-1. Ramified/resting phenotype in WT brains and activated amoeboid phenotype of microglia in transgenic AD animals was observed. (Scale bar 150 µm). **e** GFAP^+^ cells in APPPS1 animals in THA. No GFAP + cells were found in WT animals. (Scale bar 150 µm). **f** CD3^+^ T cells observed in WT-ND and APPPS1 ND and WD (Scale bar 100 µm). **g** Mean ± SD Aβ_1-42_ positive plaques load in THA in transgenic mice. **h** Mean ± SD Iba-1^+^ cells are elevated in APPPS1 animals. **i** No mean ± SD GFAP^+^ cells in WT mice, but elevated in APPPS1 mice. **j** Similar mean ± SD CD3 positive T cells in WT-ND, APPPS1-ND and APPPS1-WD group. THA = thalamus. WT-ND *n* = 2; APPPS1-ND *n* = 3; WT-WD *n* = 3; APPPS1-WD *n* = 3

**Fig. 9:**
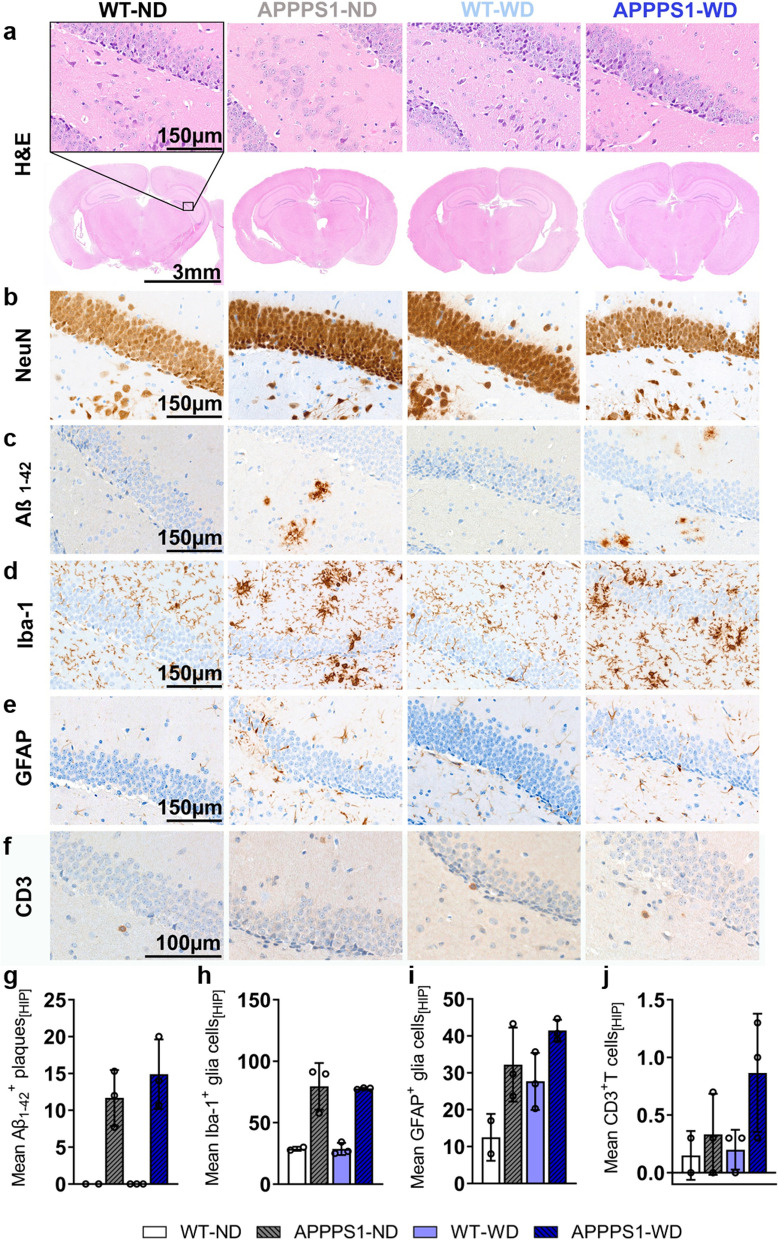
Histological analysis—Hippocampus. Histological staining results are depicted in representative images per group per staining for the hippocampus (HIP) in all groups. **a** H&E staining between groups as overview (Scale bar 3 mm) and magnification (Scale bar 150 µm). The black rectangular depicts magnification area. **b** No difference in NeuN^+^ cells between groups. **c** Aβ_1-42_ positive plaques were visible in APPPS1 animals (Scale bar 150 µm). **d** Microglia staining using Iba-1. Ramified/resting phenotype in WT brains and activated amoeboid phenotype of microglia in transgenic AD animals was observed. (Scale bar 150 µm). **e** GFAP^+^ cells in HIP of APPPS1 animals and WT-WD. (Scale bar 150 µm). **f** CD3^+^ T cells observed mainly in APPPS1 hippocampi (Scale bar 100 µm). **g** Mean ± SD Aβ_1-42_ positive plaques load in HIP in transgenic mice. **h** Mean ± SD Iba-1^+^ cells were elevated in APPPS1 animals. **i** Mean ± SD GFAP^+^ cells elevated in WT-WD and in APPPS1 mice. **j** S Higher mean ± SD CD3 positive T cells in APPPS1-WD group compared to the other groups. HIP = hippocampus. WT-ND *n* = 2; APPPS1-ND *n* = 3; WT-WD *n* = 3; APPPS1-WD *n* = 3

**Fig. 10:**
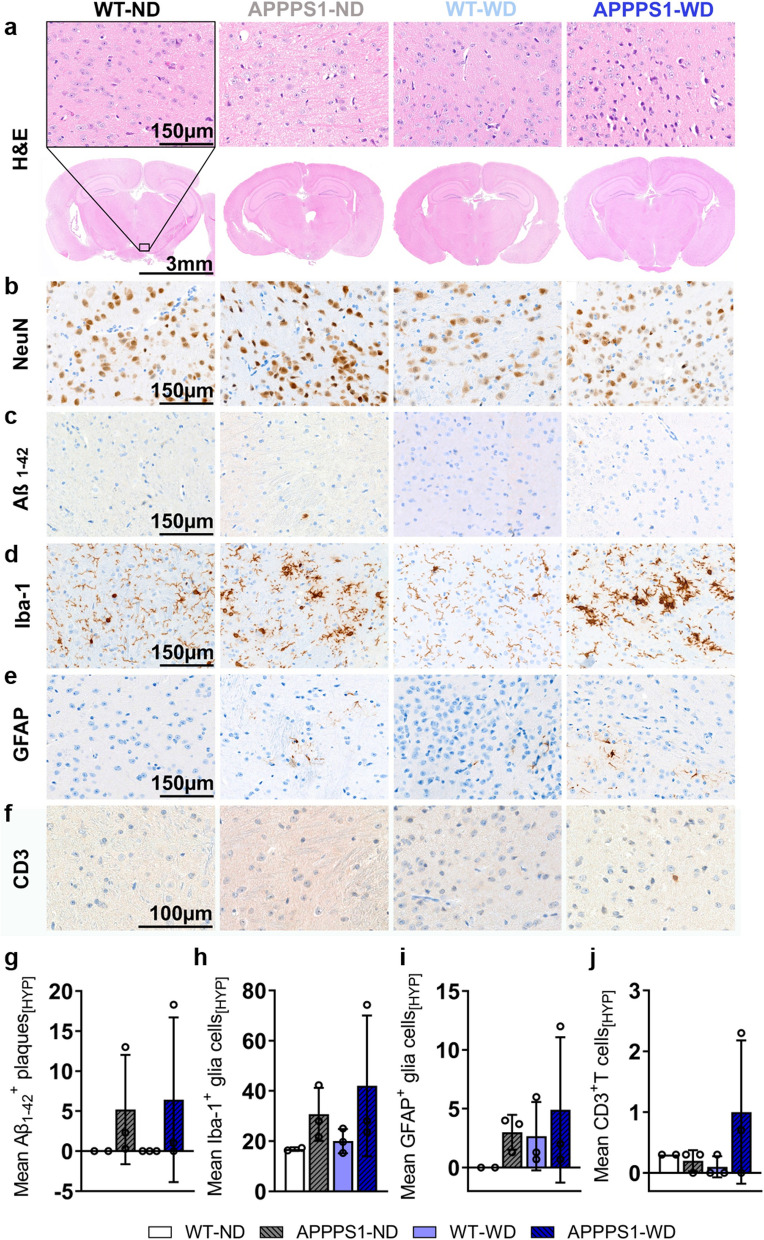
Histological analysis – Hypothalamus. Histological staining results are depicted in representative images per group per staining for the hypothalamus (HYP) in all groups. **a** H&E staining between groups as overview (Scale bar 3 mm) and magnification (Scale bar 150 µm). The black rectangular depicts magnification area. **b** No difference in NeuN^+^ cells between groups. **c** Only a few Aβ_1-42_ positive plaques were visible in APPPS1 animals with high variance between specimens (Scale bar 150 µm). **d** Microglia staining using Iba-1. Ramified/resting phenotype in WT brains and activated amoeboid phenotype of microglia in transgenic AD animals was observed. (Scale bar 150 µm) **e** GFAP^+^ cells in HYP of APPPS1 animals and WT-WD. (Scale bar 150 µm). **f** CD3^+^ T cells observed mainly in APPPS1-WD (Scale bar 100 µm). (g) Mean ± SD Aβ_1-42_ positive plaques load in HYP in transgenic mice. (h) Mean ± SD Iba-1^+^ cells were elevated in APPPS1 animals. **i** Mean ± SD GFAP^+^ cells elevated in WT-WD and in APPPS1 mice, however with high variance between animals. **j** S Higher mean ± SD CD3 positive T cells in APPPS1-WD group compared to the other groups. HYP = hypothalamus. WT-ND *n* = 2; APPPS1-ND *n* = 3; WT-WD *n* = 3; APPPS1-WD *n* = 3

**Fig. 11:**
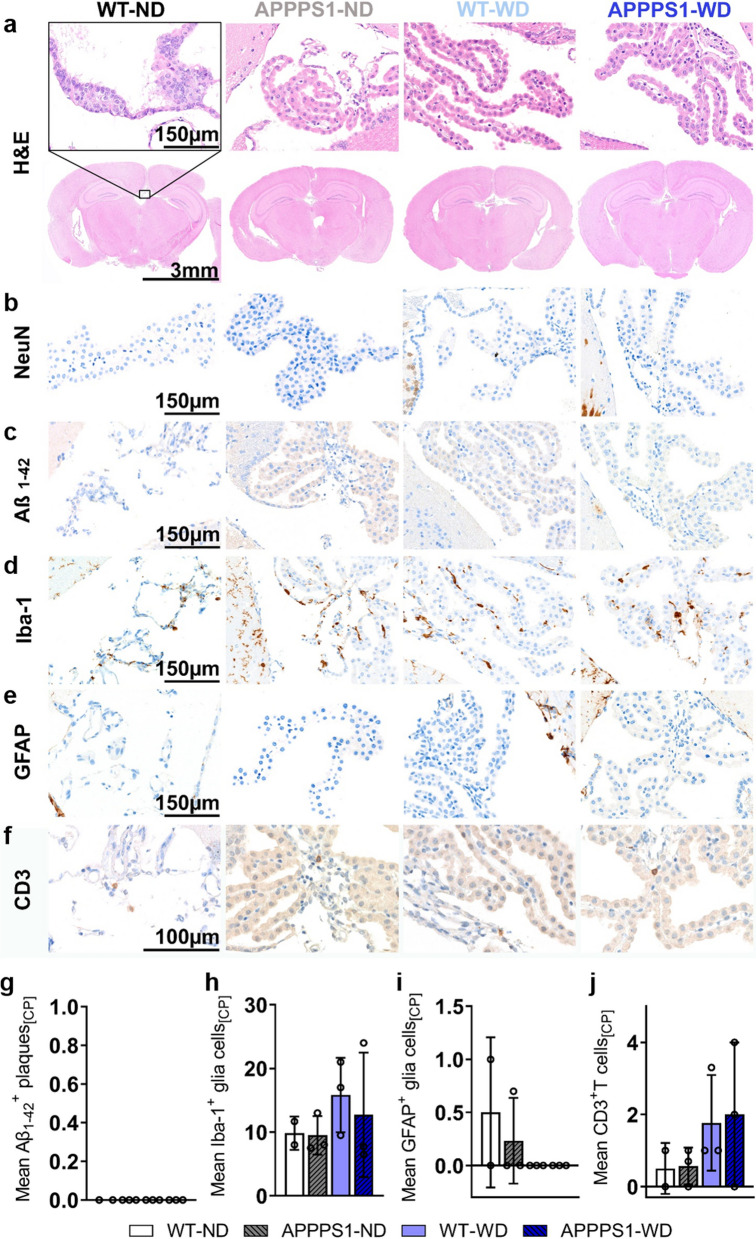
Histological analysis—Choroid plexus. Histological staining results are depicted in representative images per group per staining for the choroid plexus (CP) in all groups. **a** H&E staining between groups as overview (Scale bar 3 mm) and magnification (Scale bar 150 µm). The black rectangular depicts magnification area. **b** No NeuN^+^ cells were observed. **c** No Aβ_1-42_ positive plaques were visible in all animals (Scale bar 150 µm). **d** Microglia staining using Iba-1 (Scale bar 150 µm). e GFAP^+^ cells in CP (Scale bar 150 µm). **f** CD3^+^ T cells observed in all groups (Scale bar 100 µm). **g** No Aβ_1-42_ positive plaques were detected in CP. **h** Mean ± SD Iba-1^+^ cells were elevated in APPPS1 animals. **i** Mean ± SD GFAP^+^ cells elevated in ND groups. **j** Tendencies of higher mean ± SD CD3 positive T cells in WD group compared to the other groups. CP = choroid plexus. WT-ND *n* = 2; APPPS1-ND *n* = 3; WT-WD *n* = 3; APPPS1-WD *n* = 3

For all analyzed brains, H&E staining revealed no morphological differences between groups (Figs. 7–11). To clearly differentiate between changes between neurons and glial cells, and further examine morphological alterations, NeuN staining was performed. The NeuN IHC revealed mild differences in the APPPS1 retrosplenial areas with a smaller percentage of NeuN^+^ cells without an influence of the diet (Figs. 7–11b). No differences were detected between groups in the primary somatosensory and dorsal auditory areas (Additional file 1: Fig. S5). Amyloid plaque load in transgenic animals was high, mainly in the CTX (Fig. 7c) and THA (Fig. 8c), fewer were identified in the HIP (Fig. 9c), only a few animals showed plaques in HYP (Fig. 10c) and none were detected in choroid plexus (CP, Fig. 11c). In WD-fed groups, we could not observe that the diet increased plaque load in the investigated brain regions CTX, THA, HIP and HYP. To clarify possible differences in glial reactivity patterns upon diet and validate our in vivo results, brains were analyzed for Iba-1, a microglia marker (Figs. 7–11d and GFAP, an astrocyte marker (Figs. 7–11e). In transgenic animals, the morphology of microglia changed from a thin and ramified structure of spines to an amoeboid structure, confirming a classical activated-defined phenotype of microglia with higher cell numbers in CTX (Fig. 7), THA (Fig. 8), HIP (Fig. 9), and HYP (Fig. 10) for transgenic animals. No differences were observed between the diets for the investigated regions, consistent with the observed in vivo results. Iba-1 microglia were highly reactive in regions of high Aβ positive plaque load (Figs. 7–10c, d), whereas they were less activated in regions with few to no plaques (Fig. 8, red box). The GFAP IHC revealed that no GFAP^+^ cells were present in the CTX (Fig. 7e), THA (Fig. 8e), and HYP (Fig. 10e) of the WT animals, independently of the diet received. In contrast, the APPPS1 animals showed GFAP^+^ cells in the above-mentioned areas, without a strong influence of the diet. Interestingly, the diet did affect the number of GFAP^+^ cells in the HIP (Fig. 9e). The WT animals fed with ND showed few positive cells, while more positive cells were detected in the WT and APPPS1 animals fed with the WD, but also APPPS1 animals fed a ND. Additionally, in transgenic animals GFAP^+^ cells showed a reactive phenotype with thicker and shorter dendrites (Fig. 7e). To confirm flow cytometric T cell infiltration in the brain parenchyma, we stained for infiltrating CD3^+^ T cells and found more T cells in APPPS1-WD brains compared to the other groups in CTX (Fig. 7f) as well HIP (Fig. 9f) and HYP (Fig. 10f). Furthermore, for some animals we found a high number near the choroid plexus, the main entry site of peripheral T cells and B cells (Fig. 11), however intragroup variability was high. In wild-type animals, isolated T cells were observed independent of the diet.

## Discussion

The aging of the general population in Western countries is accompanied by an increase in the prevalence of dementia, and it is suspected that the increase in overweight and obesity exacerbates this challenge in public health. The suspected underlying link involves a general chronic inflammatory state of the patient called metaflammation, but further work is required to understand the intricacies governing cerebral metabolic disruption and unbalanced diets. In this work, we investigated such potential interaction first by using non-invasive imaging techniques to identify molecular and metabolic dysfunctions in different organs in vivo. In our experiments involving wild-type mice and a murine amyloidosis model, a surrogate for AD progression, mice were fed a WD upon manifestation of amyloid pathology, covering the preclinical early to mid-life period when pathophysiological changes can already be detected [63]. We, therefore, used an amyloidosis mouse model which is well described [45, 64] and represents an un-modifiable AD risk factor. Importantly, our model does not include age-related effects on the brain and periphery induced by the so-called inflammaging, a phenomenon proposed to be a low-grade systemic inflammatory process [65] also favoring age-related diseases like AD [66]. We chose for this study WD which is known to mimic the nutrition of Western countries with high fat and high sugar content together with simple carbohydrates and a shifted fat composition towards saturated fatty acids [67]. This led to a significant weight gain increase when fed over six months in both, male and female mice.

As the liver is one of the organs heavily affected by a high-caloric diet leading to systemic disruption and metabolic imbalance, we wanted to monitor the grade of fatty liver syndrome in WD-fed animals. Via non-invasive proton magnet resonance spectroscopy (^1^H-MRS), we detected markedly elevated lipid levels in the livers of WD-exposed mice. Similar results could also be detected in the livers of patients already after a 2-week HFD [68]. ^1^H-MRS revealed significantly higher lipid mass and fractional lipid mass together with lower levels of polyunsaturated lipids in WD compared to ND livers, which mirrors the evolution of liver composition in rats fed with a high-fat diet [69]. In another study, comparing leptin-deficient ob/ob mice to controls, a decrease in polyunsaturated lipids and an increase in saturated lipids were measured in this obesity-only model excluding dietary impact [56]. However, we could not detect differences in saturated lipids between the diets. Even though some assume a homogenous hepatic fat distribution [70], others report heterogeneous hepatic fat distribution following HFD [71, 72], making it likely that the spectra of the positioned single voxel do not reflect the whole liver condition. The changes in single hepatic lipid peaks and lipid fractions after WD endorse the impact of the diet on liver fat accumulation, even after 6 months of WD.

Metabolite analysis of plasma revealed significant differences for pyruvate, 3-hydroxybutyrate (3-HB), histidine, and isoleucine. 3-Hydroxybutyrate has been shown to function in rodents as an anti-AD drug [73] and neuroprotective agent [74]. In our study, the levels were found to be highest in ND-fed transgenic animals, but we found high levels in WD-fed animals too. Moreover, levels decline in WD-fed APPPS1 animals, which we found to have high brain glucose uptake. Together with a contradicting study, which showed 3-HB to be high in 3xTg animals but low in HFD-fed animals and was associated with glucose metabolism compensation [75], 3-HB might be an important plasma marker to indicate brain glucose metabolism changes. Administration of histidine to mice brains has been postulated to alleviate chronic effects of hypoperfusion by, among other, improving BBB integrity [76]. Even further, histidine application is associated with a neuroprotective role in AD [77]. Here, the lower levels only in APPPS1-WD animals might indicate the acceleration of detrimental processes in this group and combination with other plasma markers (3-HB, pyruvate) could be used to specify central metabolic disruptions further.

In our in vivo imaging approach, we investigated diet-induced effects on brain metabolism. By using glucose and long-chain fatty acid surrogates, we could monitor brain metabolism alterations induced by diet in wild-types and APPPS1 mice. [^18^F]FDG brain uptake was higher in WD-fed APPPS1 mice compared to the other conditions, indicating that WD may lead to higher glucose transport activity in AD transgenic animals. This regional uptake of [^18^F]FDG across the brain is a measure of glucose transport into cells and has to be distinguished from glucose oxidation, which refers to the metabolic processing of glucose once it is inside the cell. [^18^F]FDG is a glucose analog actively transported into cells primarily via glucose transporters such as GLUT1 and GLUT3, abundantly expressed at the BBB, astroglia and neurons. Therefore, [^18^F]FDG uptake as measured in our study serves as an indicator of glucose transport activity rather than direct glucose metabolism or oxidation within brain tissues. Analysis of the voxel level confirmed a whole-brain effect in APPPS1-WD animals. As studies have already shown a positive correlation between the [^18^F]FDG and [^18^F]GE-180 signal in aging wild-type mice assuming higher glucose demand due to higher glial reactivity [78], a comparison of both tracers revealed in our study no such correlations in all groups. This was complemented by Iba-1 staining in brain tissue. Although obtained using a different diet and model, these results can be compared to studies done using mice infused with human Aβ_42_ while fed an HFD over 3 months [79]. The authors could show a [^18^F]FDG hypermetabolism when diet and Aβ were combined and saw no association between TSPO signal and glucose uptake, assuming that gliosis is not the only player in diet-induced neuroinflammation. Longitudinal assessment of [^18^F]FDG brain uptake in the same transgenic model has been shown to not differ from controls in mid-age, similar to our results, but decrease with advanced age [80], assuming that the consumption of a WD can initiate hypermetabolism in this amyloid model. Interestingly, Ashraf et al. claimed that the hypermetabolic phase they observed in patients with mild cognitive impairment (MCI) might reflect a compensatory neuroplastic mechanism of neurons, which, when overstimulated, could exhaust thereby accelerating the degenerative process [81]. Thus, the high [^18^F]FDG uptake that we see in the APPPS1-WD group may represent a transient process of a neuronal compensatory response that eventually leads to neuronal death and cognitive decline as a consequence of diet-induced obesity (DIO) and/or dietary components. Even further, immunohistochemistry indicated higher numbers of GFAP + cells in the hippocampi of APPPS1-WD animals compared to the other groups assuming that the above-mentioned transient process might not be limited to neurons but could be supported by higher glucose consumption of astrocytes in this region [82]. Furthermore, metabolomic analysis revealed elevated pyruvate plasma levels in the WD-fed transgenic animals complementing the PET results. In addition, metabolomic results revealed a higher VIP (Variable Importance in Projection) score for glucose; however not significant in statistical analysis.

Many studies could show that direct or indirect (via diet) supplementation of peripheral fatty acids can activate inflammatory cascades in the brain e.g., via TLRs, and therefore induce inflammatory processes [82]. We used the long-chain fatty acid tracer [^18^F]FTHA to determine fatty acid metabolism when a continuous delivery of fatty acids is given. Brain uptake revealed indeed a diet-dependent higher fatty acid metabolism, which was independent of the genotype. In human and pig brains, [^18^F]FTHA has been shown to cross the BBB and to represent central fatty acid oxidation [83, 84]. Moreover, in patients with metabolic syndrome, [^18^F]FTHA brain uptake was higher, similar to our results. Studies propose that high levels of saturated FA could lead to a state of microglial and astrocytic reactivity [85, 86], so we compared [^18^F]GE-180 signals to the [^18^F]FTHA signals, but could not detect correlations in VOI-based or overlapping regions in voxel-wise analyses. However, no further discrimination between normal and diseased brains was found in this model using [^18^F]FTHA.

Neuroinflammation was assessed by imaging using the TSPO tracer [^18^F]GE-180which, despite its limitations (notably non-specific binding and susceptibility to genetic variations in the TSPO gene [87]) revealed higher uptake in pathology-rich regions of the transgenic brain. This increased uptake correlates with results obtained from other studies using other AD models [48, 88, 89]. In contrast with other studies showing higher glial activity after energy-rich diets (for a comprehensive overview see [90]), we could demonstrate small diet-dependent variations of GFAP^+^ cells in the hippocampus in this model. A recently published study in wild-type female mice exposed to a long-term HFD found no diet-induced [^18^F]GE-180 effects in brains supporting the obtained results in this study [91]. It is, however, also possible that the feeding duration or composition of our WD might not initiate higher glial inflammation. For instance, in a study that used two high-caloric diets in the same experimental set-up, only the diet with high lard content (60% fat) led to increased neuroinflammation, whereas the WD (40% fat) did not [92]. Furthermore, different durations of HFD seem to employ region-specific inflammatory processes in the cortex and the cerebellum of mice [93]. It is important to note that the TSPO tracer, [^18^F]GE-180 has been the subject of an extensive debate as several studies have shown that the tracer only has notable uptake when the integrity of the BBB is sufficiently affected by the pathology [94]. Our results show higher brain uptake of [^18^F]GE-180 in transgenic mice compared to wild-types for the pathology-rich cortex, which could be related to an altered BBB in WD-fed APPPS1 mice. In DIO rats fed a WD, an elevated BBB permeability was not observed earlier than 90 days, suggesting a gradual BBB breakdown [95]. Other studies report no increased permeability [93, 96]. However, in our study, the Western diet might act as an additive factor for BBB permeability in amyloid-prone animals by disturbing brain metabolic balance. Further investigations need to validate this hypothesis. Systemic and central alterations caused by the chronic consumption of a high-caloric diet or the AD pathology per se might affect tracer uptake into the brain. By determining regional cerebral blood flow (rCBF) no changes in perfusion were reported for our AD model [97] and in mice fed a WD for 12 weeks [98], even after HFD for six months, lower perfusion was measured [96]. However, other studies report no increased permeability [93]. In DIO rats fed a WD, an elevated BBB permeability was not observed earlier than 90 days, suggesting a gradual BBB breakdown [95]. In our study, WD might act as an additive factor for BBB permeability in amyloid-prone animals by disturbing brain metabolic balance. However, further investigations need to clarify this hypothesis.

The neuroinflammatory concept is constantly under revision and extensive work in this field is ongoing [99], supporting evidence in addition to the initial amyloid cascade hypothesis, that systemic alterations act as neuroinflammatory drivers by activating inflammatory processes e.g. by immune cell infiltration and activation. To determine immune cell involvement in the brain, we examined infiltration of innate immune cells as they have been shown to invade brains after HFD treatment [39] as well as in AD-prone mice models [42]. Except for lower Ly6G^+^ populations, no higher infiltration of innate immune cells was detected, but we could find a higher number of CD3^+^ cells in the group of APPPS1-WD whose infiltration we validated via histological staining. Further discrimination into CD3^+^CD8^−^ T helper cells and CD3^+^CD8^+^ cytotoxic T cells revealed significantly higher cytotoxic T cells in the WD-fed transgenic animals compared to the wild-types. In patients of advanced stages of AD, a study could also find a higher CD3^+^ T cell population in brains, which were CD8 positive [100]. A recent study in male rats reported higher CD8^+^ T cell infiltration in aged rats fed a three-day HFD but no higher number of CD4^+^ T cells [101]. We could not identify T cells near plaques in our model, which is in line with other reports that have shown T cells to be present in mouse models of AD, but could not see interaction with the plaques or tau pathology [102, 103]. The role T cells play in neurodegenerative disease and which mechanisms the infiltrating T cells initiate once they reside in the parenchyma is still discussed. Several studies point towards a neuro-protective role in AD mouse models [36, 44], while others report detrimental effects [43]. Further discrimination of T cell subtypes in our study, could show a polarization towards an effector memory or activated effector phenotype of both CD8^−^ and CD8^+^ T cells in the WD group. The chronic metabolic inflammation in organs like adipose tissue, which display a constant pro-inflammatory burden for the body, might facilitate the activation of naïve T cells in immune compartments of the periphery before they enter the CNS [104, 105]. As we could detect positive associations only between distinct immune cells populations and PET tracers, further studies are needed to draw conclusive relationships and clarify whether the T cells are polarized by signals from the periphery or via CNS internal signals and to which extent they disturb CNS homeostasis and accelerate inflammatory processes needs to be further clarified.

The investigation of the metaflammatory condition in white adipose tissue (WAT) in the periphery has proven to correspond to studies describing macrophage recruitment and polarization towards pro-inflammatory status in inflamed WAT [12]. Although we found higher B cell populations in WD-WAT, which contribute to systemic inflammation by modulating T cells and macrophages [106–108], no significant changes in T cell populations were detected in our model. Overall, results clearly show disruption of innate immune cell infiltrates in WAT of WD-fed animals with minor changes in T cell populations. To our knowledge, we were the first to compare WAT immune cells of APPPS1 and wild-types, which showed no differences for both diets. Further experiments to distinguish T cell phenotypes would be helpful to examine the impact of the WD on the T cells in WAT.

Imaging methods prove indispensable for AD diagnosis, being generally non-invasive and relatively well-tolerated by patients. In AD patients, [^18^F]FDG is used as a reliable marker to assess cognitive impairment and differentiating dementia types [109, 110]. [^18^F]FDG PET can be used to detect regions which will possibly develop AD in non-demented individuals [111, 112], an advantage to amyloid tracers which detect pathology but cannot assess cognitive decline [110, 113]. In view of the fact that not only the number of Alzheimer's patients will increase [114], but also the number of people suffering from obesity [1], the differentiated consideration of established methods is becoming increasingly important. Our results offer a new perspective to clinical studies interpreting [^18^F]FDG uptake in people potentially at risk of Alzheimer's disease due to Aβ depositions but not cognitively impaired. In overweight individuals, [^18^F]FDG uptake may differ from current state of studies, especially in the case of pre-existing pathology. As discussed above, our results may indicate an early stage of preclinical AD, demonstrating compensatory mechanisms that may be followed by cognitive impairments [81]. Therefore, more frequent monitoring may be necessary during clinical observations. Interestingly, with view of the other markers used in this study, neither [^18^F]FTHA nor [^18^F]GE-180 have been correlated to the glucose uptake seen in the APPPS1-WD group. A recently published clinical PET study using second-generation TSPO tracer [^11^C]PBR28 and amyloid tracer [^11^C]PiB could not show any correlation between amyloid burden and neuroinflammation similar to our findings. Moreover, they found no association between midlife insulin resistance or metaflammation and neuroinflammation but positive association between metabolic risk factors (high BMI and insulin resistance) and TSPO in brain regions first affected by Aβ accumulation in AD assuming dynamic neuroinflammatory processes in the course of AD and lifestyle.

We are aware that our study includes limitations, which are discussed in the following paragraph. For the in vivo studies, we chose to compare the SUV to correct for the significant weight changes between diet groups. We chose to not compare SUV ratios due to the lack of an adequate reference region in our project. Mostly the cerebellum is used as a pseudo-reference region, but its uptake changed significantly between WT under the control diet and AD mice under WD for [^18^F]FDG and [^18^F]FTHA. That the cerebellum is affected by the diet has already been observed in another study [93]. When interpreting [^18^F]FDG results from different studies, several factors should be considered. The chosen AD models seem to have a significant impact on the outcome of studies looking at the brain metabolism with a decreased [80, 115], increased [88, 116], or no different brain [^18^F]FDG uptake [117]. Our results show higher brain uptake of [^18^F]GE-180 in transgenic mice compared to wild-types for the pathology-rich cortex, which could be related to an altered BBB in WD-fed APPPS1 mice as discussed previously. While [^18^F]GE-180 is a promising high-affinity TSPO PET tracer, potentially offering improved sensitivity in detecting neuroinflammation [118, 119], TSPO imaging is still challenging. Nonspecific binding and susceptibility to genetic variations in the TSPO gene still impact [^18^F]GE-180 and similar tracer’s reliability [94, 120]. Ongoing research aims to optimize TSPO tracers enhancing the credibility of neuroinflammation assessments through PET imaging but the development of alternative inflammatory tracers is of enormous importance for future studies in neurological disorders [121]. The low power for sex differences of diet-induced effects in the AD model did not allow us to obtain conclusive results, but should be considered for future experiments as it has very recently been shown that higher body-mass-index is associated with elevated microglial activation in female patients, but not in male [122] and that also in human AD patients, metabolomics analysis results can be triggered by sex differences [123].

In this study, we propose that in AD-prone brains, further mechanisms are triggered by a WD beyond the classical microglial neuroinflammation. Moreover, we encourage further studies to examine the relation of T cells and brain glucose metabolism in AD, as both were elevated in the amyloidosis model after the WD.

### Supplementary Information

**Additional file 1:** **Table S1. **Comparison between the content of the normal diet and the Western diet. Changes indicate higher or lower portions in WD compared to ND for each compound. **Table S2. **Group distribution of experimental animals (*n*) per sex for weight, experiments and per group. Weight measurement started for ND-group at various time points (see comment). **Table S3. **Lipids of different chain lengths and lipid compositions are listed. Mean values, standard deviation (SD), and animal numbers (*n*) for ND and WD are depicted. *According to Ye et al. [56]. **Figure S1. **Gating strategy for myeloid flow cytometry antibody panel. For each immune cell population the respective antibodies are displayed on the Y- and X-axis. Gating is shown as a black outline within the plots. **Figure S2. **Gating strategy for T cell flow cytometry antibody panel. For each immune cell population the respective antibodies are displayed on the Y- and X-axis. Gating is shown as a black outline within the plots. Gating for Tregs applies only to CD8^-^ T cell populations. **Figure S3.** Typical MRS spectrum of the liver fat composition highlighting the main lipid components of interest shown in Fig. 2 and Table S3. **Figure S4** Mean ± SD blood glucose levels measured before [^18^F]FDG imaging for all 4 groups did not differ. WT-ND *n* = 11, APPPS1-ND *n* = 7, WT-WD *n* = 7, APPPS1-WD *n*= 8, post hoc Holm-Sidak corrected for multiple comparisons. **Table S4. **Overview table with results from NMR raw spectral analysis: annotated metabolite names, their corresponding signals in the ^1^H spectrum (indicative multiplicity and experimentally monitored chemical shift), and also a report of averaged (means) normalized concentrations per metabolite per group with a given standard deviation (SD). S, singlet; D, doublet, D–D, doublet of doublets, D–D–D, doublet of doublets of doublets; T, triplet; Q, quartet; M, multiplet. A.u. der. mM, arbitrary units derived from millimolar concentration values. Negative values are a result of pareto scaling. **Table S5. **Metabolomics data changes overview in three mice group comparisons (**A** APPPS1-ND vs. WT-ND; **B** WT-WD vs. WT-ND; **C** APPPS1-WD vs. WT-ND) checked against the control ND-WT mice. VIP (Variable Importance in Projection) scores are depicted for each group. Statistical significance: *p < 0.05, **p < 0.01, ***p < 0.001. Non-significant changes (by t-test) were not labeled. **Table S6. **Pearson’s correlations of whole brain uptake (SUV) of PET tracers [^18^F]FDG, [^18^F]FTHA, [^18^F]GE-180 and relevant immune cells in brains (percentage of viable cells) for all experimental groups. For each comparison the correlation coefficient r, r squared R^2^, *p*-value and number of compared values *n* are shown. Statistical significance: *p < 0.05, **p < 0.01, ***p < 0.001. **Figure S5.** Percentage of positive NeuN cells in cortex (CTX) areas: retrosplenial area, primary somatosensory area and dorsal auditory area. WT-ND *n* = 2; APPPS1-ND *n* = 3; WT-WD *n* = 3; APPPS1-WD *n* = 3.

## Data Availability

The datasets and material generated during and/or analyzed during the current study are available from the corresponding author upon reasonable request.
