# Supplementary material for: Western diet increases brain metabolism and adaptive immune responses in a mouse model of amyloidosis
Source: J Neuroinflammation. 2024 May 14;21:129. doi: 10.1186/s12974-024-03080-0 (PMC11092112; doi:10.1186/s12974-024-03080-0)
Supplement: Supplementary file 1 — Additional file 1: Table S1. Comparison between the content of the normal diet and the Western diet. Changes indicate higher or lower portions in WD compared to ND for each compound. Table S2. Group distribution of experimental animals (n) per sex for weight, experiments and per group. Weight measurement started for ND-group at various time points (see comment). Table S3. Lipids of different chain lengths and lipid compositions are listed. Mean values, standard deviation (SD), and animal numbers (n) for ND and WD are depicted. *According to Ye et al. [56]. Figure S1. Gating strategy for myeloid flow cytometry antibody panel. For each immune cell population the respective antibodies are displayed on the Y- and X-axis. Gating is shown as a black outline within the plots. Figure S2. Gating strategy for T cell flow cytometry antibody panel. For each immune cell population the respective antibodies are displayed on the Y- and X-axis. Gating is shown as a black outline within the plots. Gating for Tregs applies only to CD8- T cell populations. Figure S3. Typical MRS spectrum of the liver fat composition highlighting the main lipid components of interest shown in Fig. 2 and Table S3. Figure S4 Mean ± SD blood glucose levels measured before [18F]FDG imaging for all 4 groups did not differ. WT-ND n = 11, APPPS1-ND n = 7, WT-WD n = 7, APPPS1-WD n= 8, post hoc Holm-Sidak corrected for multiple comparisons. Table S4. Overview table with results from NMR raw spectral analysis: annotated metabolite names, their corresponding signals in the 1H spectrum (indicative multiplicity and experimentally monitored chemical shift), and also a report of averaged (means) normalized concentrations per metabolite per group with a given standard deviation (SD). S, singlet; D, doublet, D–D, doublet of doublets, D–D–D, doublet of doublets of doublets; T, triplet; Q, quartet; M, multiplet. A.u. der. mM, arbitrary units derived from millimolar concentration values. Negative values are a result of pa [file 12974_2024_3080_MOESM1_ESM.doc]

**Additional Material**

**Additional Table 1**

Comparison between the content of the normal diet and the Western diet. Changes indicate higher or lower portions in WD compared to ND for each compound.

**Additional Table 2**

Group distribution of experimental animals (*n*) per sex for weight, experiments and per group. Weight measurement started for ND-group at various time points (see comment).

| **Group** | **WT- ND (*n*)** | | **APPPS1-ND (*n*)** | | **WT-WD (*n*)** | | **APPPS1-WD (*n*)** | | **comment** |
| --- | --- | --- | --- | --- | --- | --- | --- | --- | --- |
| *Experiment* | female | male | female | male | female | male | female | male |  |
| *weight* | TP_0_=1  TP_4_=3  TP_5_=4 | TP_0_=3  TP_4_=4  TP_5_=7 | TP_0_=3  TP_4_=3  TP_5_=4 | TP_0_=1  TP_4_=3  TP_5_=5 | TP_0_=6 | TP_0_=6 | TP_0_=6 | TP_0_=6 | For ND:  *n*=8 at time point 0 (start)  *n*=13 at time point 4 weeks  *n*= 20 at time point 5 weeks |
| *Metabolomics* | 4 | 7 | 4 | 4 | 6 | 4 | 5 | 4 | Animal numbers can be found in methods and in figure legends |
| *^1^H-MRS* | 3 | 3 | 1 | 2 | 2 | 5 | 2 | 3 |
| *[^18^F]FDG* | 4 | 6 | 4 | 3 | 3 | 4 | 3 | 5 |
| *[^18^F]FTHA* | 3 | 5 | 4 | 4 | 3 | 5 | 3 | 4 |
| *[^18^F]GE-180* | 3 | 5 | 4 | 3 | 4 | 5 | 5 | 5 |
| *Flow cytometry brain* | 4 | 7 | 4 | 4 | 6 | 4 | 5 | 4 |
| *Flow cytometry WAT* | 4 | 7 | 4 | 4 | 6 | 4 | 5 | 2 |

**Additional Table 3**

Lipids of different chain lengths and lipid compositions are listed. Mean values, standard deviation (SD), and animal numbers (*n*) for ND and WD are depicted. *according to Ye et al. 2012

| *Lipids* | *ND* | | | *WD* | | | *Lipids-associated ^1^H of the spectra** |
| --- | --- | --- | --- | --- | --- | --- | --- |
|  | **mean** | **SD** | ***n*** | **mean** | **SD** | ***n*** |  |
| *Lip09* | 0,004 | 0,002 | 10 | 0,038 | 0,023 | 13 | CH_3_-(CH_2_)n- |
| *Lip13* | 0,021 | 0,011 | 11 | 0,240 | 0,145 | 13 | -(CH_2_)n- |
| *Lip16* | 0,003 | 0,002 | 8 | 0,020 | 0,013 | 13 | -CH_2_-O-CO-CH_2_-CH_2_- |
| *Lip21* | 0,004 | 0,002 | 10 | 0,036 | 0,021 | 13 | -CH_2_-CH_2_-CH=CH- |
| *Lip23* | 0,002 | 0,001 | 8 | 0,023 | 0,017 | 13 | CH_2_-O-CO-CH_2_-CH_2_- |
| *Lip28* | 0,001 | 0,001 | 7 | 0,003 | 0,004 | 7 | -CH=CH-CH_2_-CH=CH- |
| *Lip41* | 0,001 | 0,001 | 5 | 0,006 | 0,004 | 13 | -CH_2_-O-C(O)-CH_2_-CH_2_- |
| *Lip43* | 0,001 | 0,001 | 6 | 0,006 | 0,004 | 13 | CH_2_-O-C(O)-CH_2_-CH_2_- |
| *Lip53+Lip52* | 0,003 | 0,001 | 8 | 0,022 | 0,013 | 13 | -CH=CH- and >CH-CH_2_-O-C(O)-CH_2_-CH_2_- |
| *Water* | 0,049 | 0,038 | 11 | 0,036 | 0,014 | 13 |  |
|  |  |  |  |  |  |  |  |
| *lipid mass* | 0,357 | 0,146 | 9 | 3,347 | 2,004 | 13 |  |
| *fLM* | 0,484 | 0,187 | 9 | 0,8771 | 0,093 | 13 |  |
| *SL* | 8,315 | 0,542 | 9 | 9,510 | 1,857 | 13 |  |
| *fUL* | 0,852 | 0,384 | 9 | 0,7426 | 0,098 | 13 |  |
| *fSL* | 0,273 | 0,083 | 8 | 0,258 | 0,098 | 13 |  |
| *fPUL* | 0,295 | 0,010 | 7 | 0,116 | 0,105 | 7 |  |
| *fMUL* | 0,563 | 0,395 | 7 | 0,625 | 0,150 | 7 |  |
| *MCL* | 15,493 | 1,562 | 9 | 16,684 | 2,412 | 13 |  |


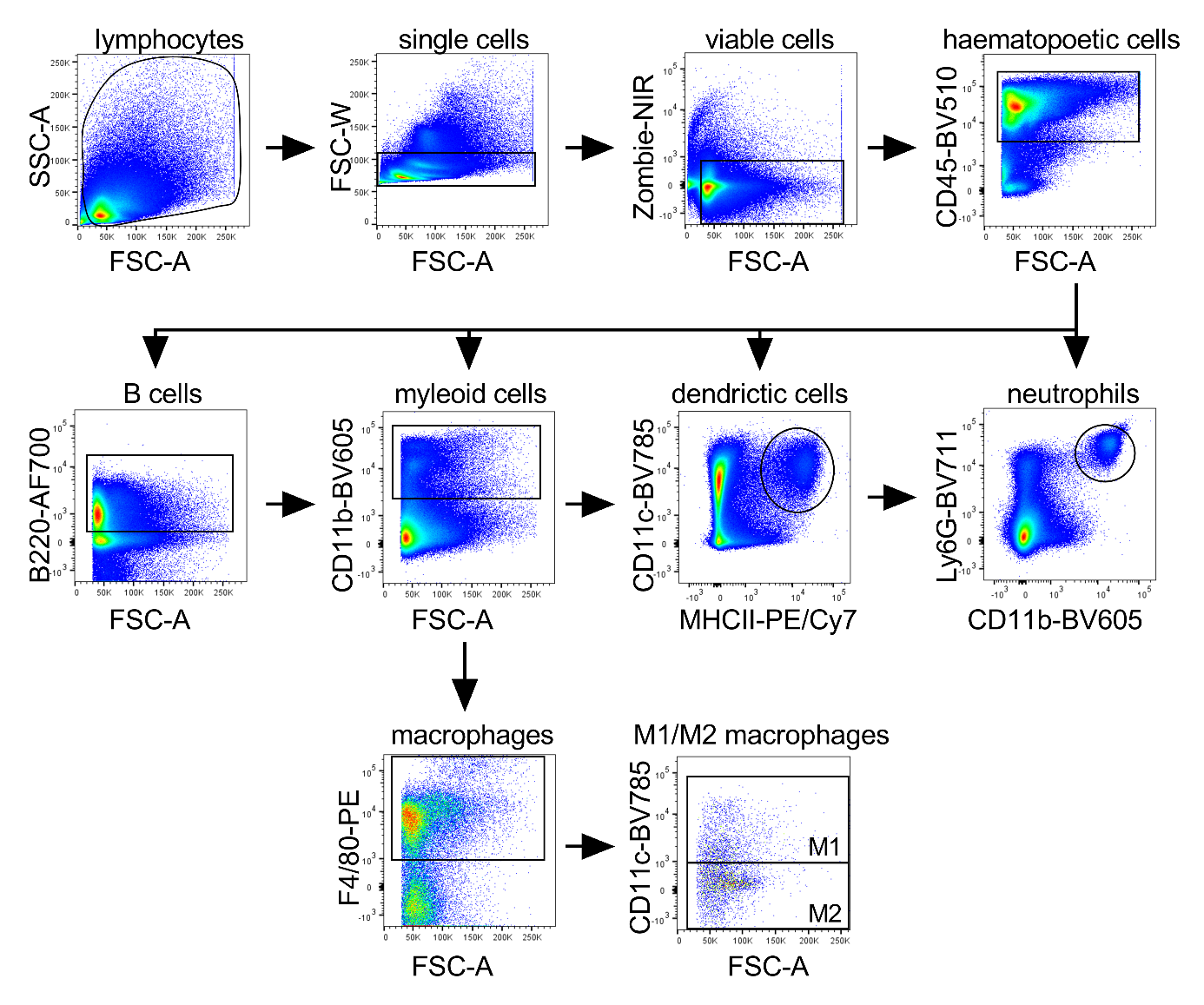
**Additional Fig.1**

Gating strategy for myeloid flow cytometry antibody panel. For each immune cell population the respective antibodies are displayed on the Y- and X-axis. Gating is shown as a black outline within the plots.


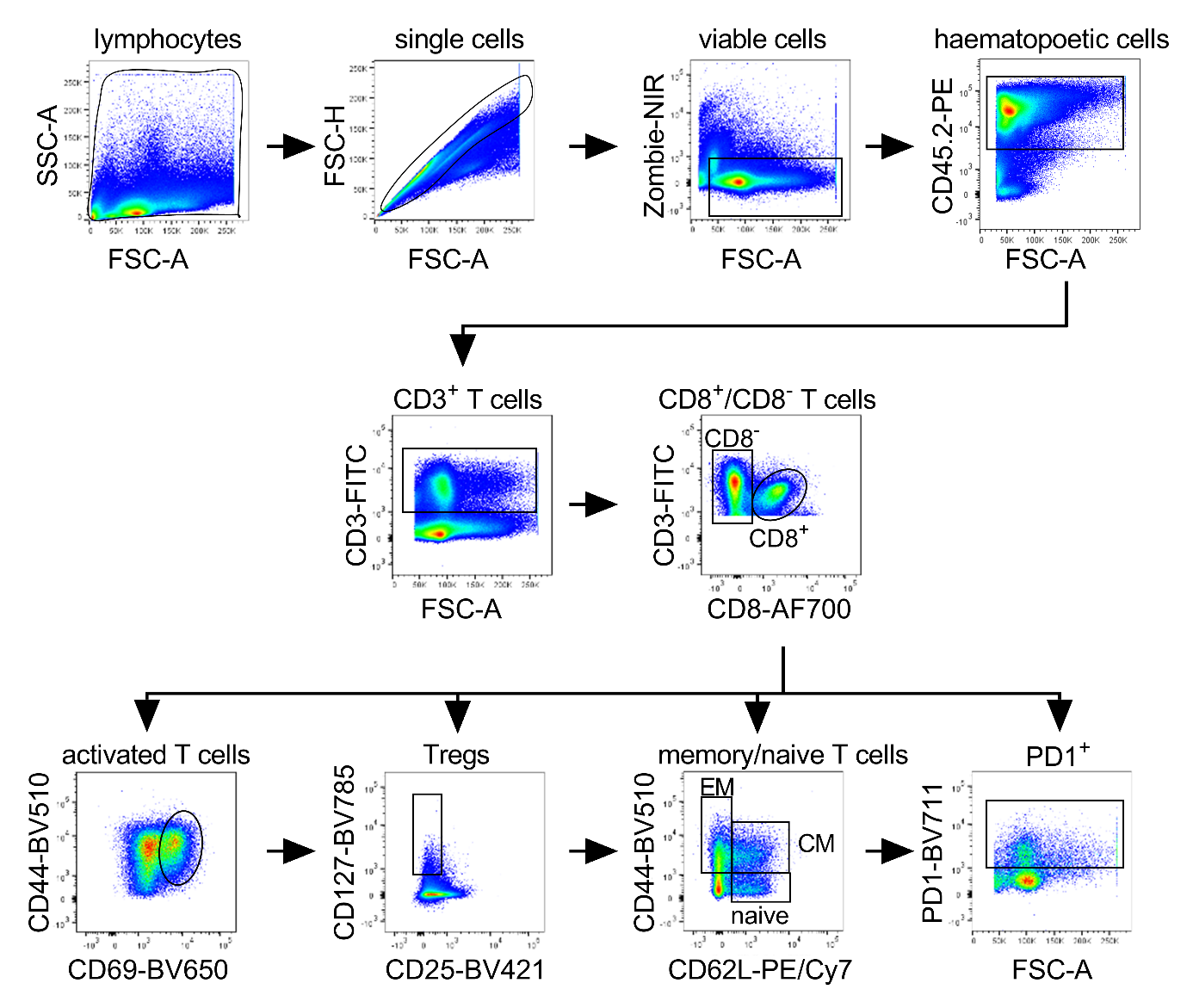
**Additional Fig. 2**

Gating strategy for T cell flow cytometry antibody panel. For each immune cell population the respective antibodies are displayed on the Y- and X-axis. Gating is shown as a black outline within the plots. Gating for Tregs applies only to CD8^-^ T cell populations.

**Additional Fig. 3**

**
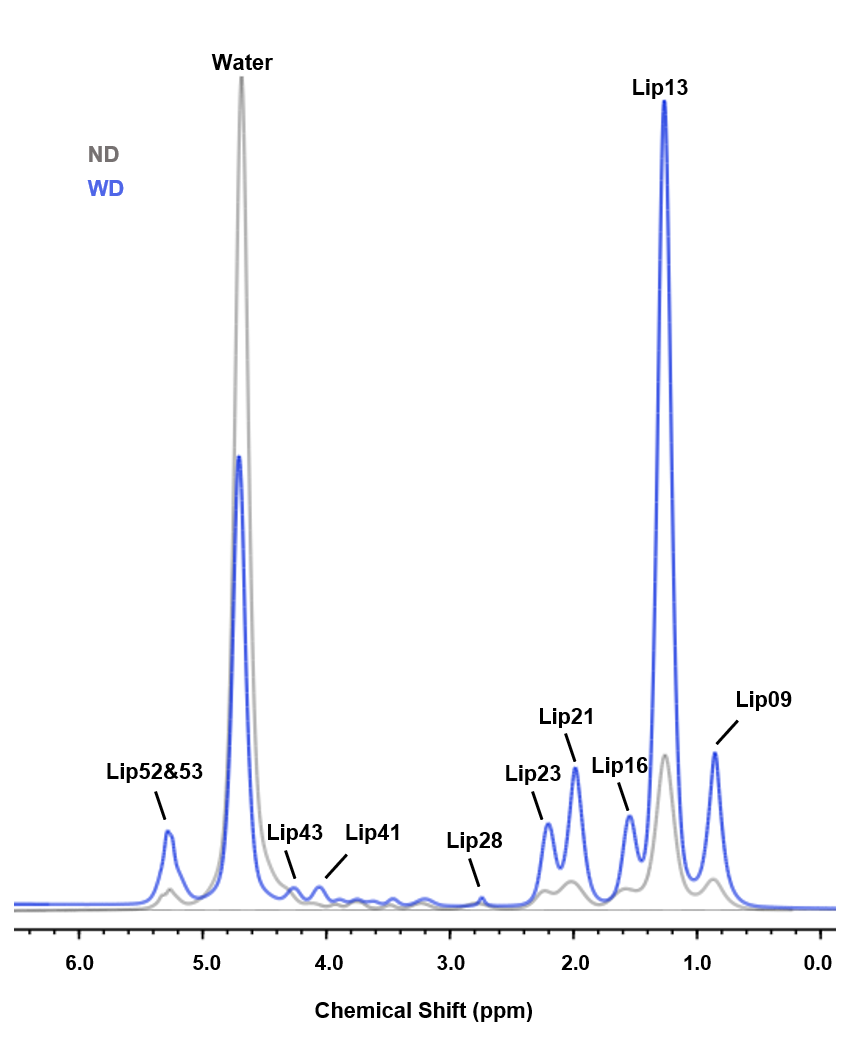
**

Typical MRS spectrum of the liver fat composition highlighting the main lipid components of interest shown in Figure 2 and Additional Table 3.

**Additional Table 4**

Overview table with results from NMR raw spectral analysis: annotated metabolite names, their corresponding signals in the ^1^H spectrum (indicative multiplicity and experimentally monitored chemical shift), and also a report of averaged (means) normalized concentrations per metabolite per group with a given standard deviation (SD). S – singlet; D – doublet, D-D - doublet of doublets, D-D-D - doublet of doublets of doublets; T – triplet; Q – quartet; M – multiplet. A.u. der. mM – arbitrary units derived from millimolar concentration values. Negative values are a result of pareto scaling.

| *Metabolite* | *NMR signals* | | *APPPS1 -ND* | *WT -WD* | *APPPS1 -WD* | *WT -ND* |
| --- | --- | --- | --- | --- | --- | --- |
|  | **Multiplicity** | Exp. chemical shift, ppm | Mean (avg) ±SD,  Norm. conc.  a.u. der. mM | Mean (avg) ±SD,  Norm. conc.  a.u. der. mM | Mean (avg) ±SD,  Norm. conc.  a.u. der. mM | Mean (avg) ±SD,  Norm. conc.  a.u. der. mM |
| *3-HB* | **D-D & D-D** | 2.35 & 2.25 | 0.052±0.07 | 0.05±0.09 | -0.013±0.06 | -0.073±0.06 |
| *Acetate* | **S** | 1.88 | 0.063±0.07 | -0.019±0.04 | 0.008±0.08 | -0.035±0.18 |
| *Formate* | **S** | 8.42 | -0.085±0.08 | -0.015±0.12 | 0.059±0.19 | 0.026±0.22 |
| *Isobutyrate* | **D** | 1.03 | 0.003±0.02 | 0.007±0.02 | 0.003±0.03 | -0.012±0.04 |
| *Alanine* | **D** | 1.44 | -0.007±0.15 | -0.037±0.15 | 0.109±0.13 | -0.051±0.23 |
| *Glutamine* | **M** | 2.41 | 0.052±0.18 | -0.048±0.13 | 0.01±0.23 | -0.003±0.2 |
| *Glycine* | **S** | 3.52 | 0±0.1 | -0.047±0.1 | 0.069±0.19 | -0.013±0.09 |
| *Histidine* | **S** | 7.02 | -0.002±0.04 | 0.02±0.05 | -0.057±0.07 | 0.03±0.04 |
| *Isoleucine* | **D** | 0.97 | 0.019±0.05 | 0.036±0.08 | 0.01±0.04 | -0.054±0.04 |
| *Leucine* | **T** | 0.92 | -0.02±0.05 | 0.029±0.1 | -0.013±0.03 | -0.002±0.05 |
| *Lysine* | **M** | 1.69 | 0.06±0.14 | -0.042±0.1 | -0.053±0.05 | 0.038±0.14 |
| *Methionine* | **T** | 2.60 | -0.003±0.05 | -0.003±0.05 | 0.002±0.04 | 0.003±0.05 |
| *Phenylalanine* | **D (D-D)** | 7.29 | -0.021±0.02 | 0.02±0.06 | -0.021±0.03 | 0.015±0.05 |
| *Proline* | **M** | 2.00 | 0.037±0.09 | -0.032±0.13 | -0.01±0.09 | 0.01±0.1 |
| *Tyrosine* | **D (D-D-D)** | 6.86 | -0.027±0.05 | 0.027±0.06 | -0.032±0.06 | 0.022±0.06 |
| *Valine* | **D & D** | 1.00 & 0.95 | 0±0.12 | -0.002±0.12 | 0.003±0.08 | 0±0.07 |
| *Citrate* | **D & D** | 2.62 & 2.49 | -0.026±0.14 | 0.026±0.1 | -0.05±0.09 | 0.036±0.2 |
| *Fumarate* | **S** | 6.48 | -0.007±0.01 | -0.016±0.03 | 0.006±0.02 | 0.015±0.04 |
| *Succinate* | **S** | 2.36 | -0.034±0.01 | -0.034±0.01 | -0.034±0.02 | 0.084±0.23 |
| *Creatine* | **S & S** | 3.89 & 3.00 | 0.001±0.05 | -0.008±0.06 | -0.036±0.04 | 0.035±0.09 |
| *Creatinine* | **S** | 3.02 | 0.006±0.04 | 0.008±0.02 | -0.02±0.03 | 0.005±0.04 |
| *Glucose* | **D** | 5.20 | -0.223±0.55 | 0.339±0.57 | 0.181±0.53 | -0.294±0.94 |
| *Lactate* | **Q** | 4.07 | -0.23±0.28 | -0.018±0.29 | 0.207±0.46 | 0.014±0.63 |
| *Pyruvate* | **S** | 2.33 | -0.062±0.02 | 0.006±0.09 | 0.102±0.05 | -0.044±0.04 |

**Additional Table 5**

Metabolomics data changes overview in three mice group comparisons (A - APPPS1-ND vs. WT-ND; B - WT-WD vs. WT-ND; C - APPPS1-WD vs. WT-ND) checked against the control ND-WT mice. VIP (Variable Importance in Projection) scores are depicted for each group. Statistical significance: **p* < 0.05, ***p* < 0.01, ****p* < 0.001. Non-significant changes (by t-test) were not labeled.

| *Description* |  | *APPPS1-ND / WT-ND* | | *WT-WD / WT-ND* | | *APPPS1-WD / WT-ND* | |
| --- | --- | --- | --- | --- | --- | --- | --- |
|  |  | **A** | VIP | **B** | VIP | **C** | VIP |
| ***Main PLS-DA Component (1)*** | **Explained Variance, %** |  | 29.30% |  | 60.50% |  | 53.70% |
|  | *Metabolite* |  |  |  |  |  |  |
| ***Ketone body*** | **3-Hydroxybutyrate** | **↑**** | **1.6** | **↑**** | **0.9** | **↑*** | **0.5** |
| ***Short-chain fatty acids*** | Acetate | ↑ | 1.3 | ↑ | 0.1 | **↑** | 0.3 |
|  | Formate | ↓ | 1.5 | ↓ | 0.3 | **↑** | 0.3 |
|  | Isobutyrate | ↑ | 0.2 | **↑** | 0.1 | **↑** | 0.1 |
| ***Amino acids*** | Alanine | ↑ | 0.6 | **↑** | 0.1 | **↑** | 1.3 |
|  | Glutamine | ↑ | 0.7 | ↓ | 0.3 | **↑** | 0.1 |
|  | Glycine | ↑ | 0.2 | ↓ | 0.2 | **↑** | 0.7 |
|  | **Histidine** | ↓ | 0.4 | ↓ | 0.1 | **↓**** | **0.7** |
|  | **Isoleucine** | **↑**** | **1.0** | **↑**** | **0.6** | **↑**** | **0.5** |
|  | Leucine | ↓ | 0.2 | **↑** | 0.2 | ↓ | 0.1 |
|  | Lysine | ↑ | 0.3 | ↓ | 0.6 | ↓ | 0.7 |
|  | Methionine | ↓ | 0.1 | ≈ | < 0.1 | ≈ | < 0.1 |
|  | Phenylalanine | ↓ | 0.5 | ≈ | < 0.1 | ↓ | 0.3 |
|  | Proline | ↑ | 0.4 | ↓ | 0.3 | ↓ | 0.2 |
|  | Tyrosine | ↓ | 0.6 | ≈ | < 0.1 | ↓ | 0.4 |
|  | Valine | ≈ | < 0.1 | ≈ | < 0.1 | ≈ | < 0.1 |
| ***Krebs cycle (TCA)*** | Citrate | ↓ | 0.8 | ↓ | 0.1 | ↓ | 0.7 |
|  | Fumarate | ↓ | 0.3 | ↓ | 0.2 | ↓ | 0.1 |
|  | Succinate | ↓ | 1.5 | ↓ | 0.9 | ↓ | 0.9 |
| ***Creatine metabolism*** | **Creatine** | ↓ | 0.4 | ↓ | 0.3 | **↓*** | **0.6** |
|  | Creatinine | ≈ | < 0.1 | ≈ | < 0.1 | ↓ | 0.2 |
| ***Glycolysis and lactate production*** | Glucose | ↑ | 0.9 | **↑** | 4.6 | **↑** | 3.8 |
|  | Lactate | ↓ | 3.2 | ↓ | 0.2 | **↑** | 1.5 |
|  | **Pyruvate** | ↓ | 0.2 | **↑** | 0.4 | **↑***** | **1.2** |

**Additional Fig. 4**


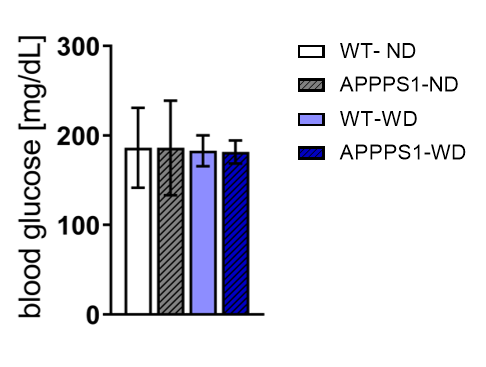


Mean ± SD blood glucose levels measured before [^18^F]FDG imaging for all 4 groups did not differ. WT-ND *n* = 11, APPPS1-ND *n* = 7, WT-WD *n* = 7, APPPS1-WD *n*= 8, post hoc Holm-Sidak corrected for multiple comparisons.

**Additional Table 6**

Pearson’s correlations of whole brain uptake (SUV) of PET tracers [^18^F]FDG, [^18^F]FTHA, [^18^F]GE-180 and relevant immune cells in brains (percentage of viable cells) for all experimental groups. For each comparison the correlation coefficient r, r squared R^2^, *p*-value and number of compared values *n* are shown. Statistical significance: **p* < 0.05, ***p* < 0.01, ****p* < 0.001

**Additional Figure 5:**


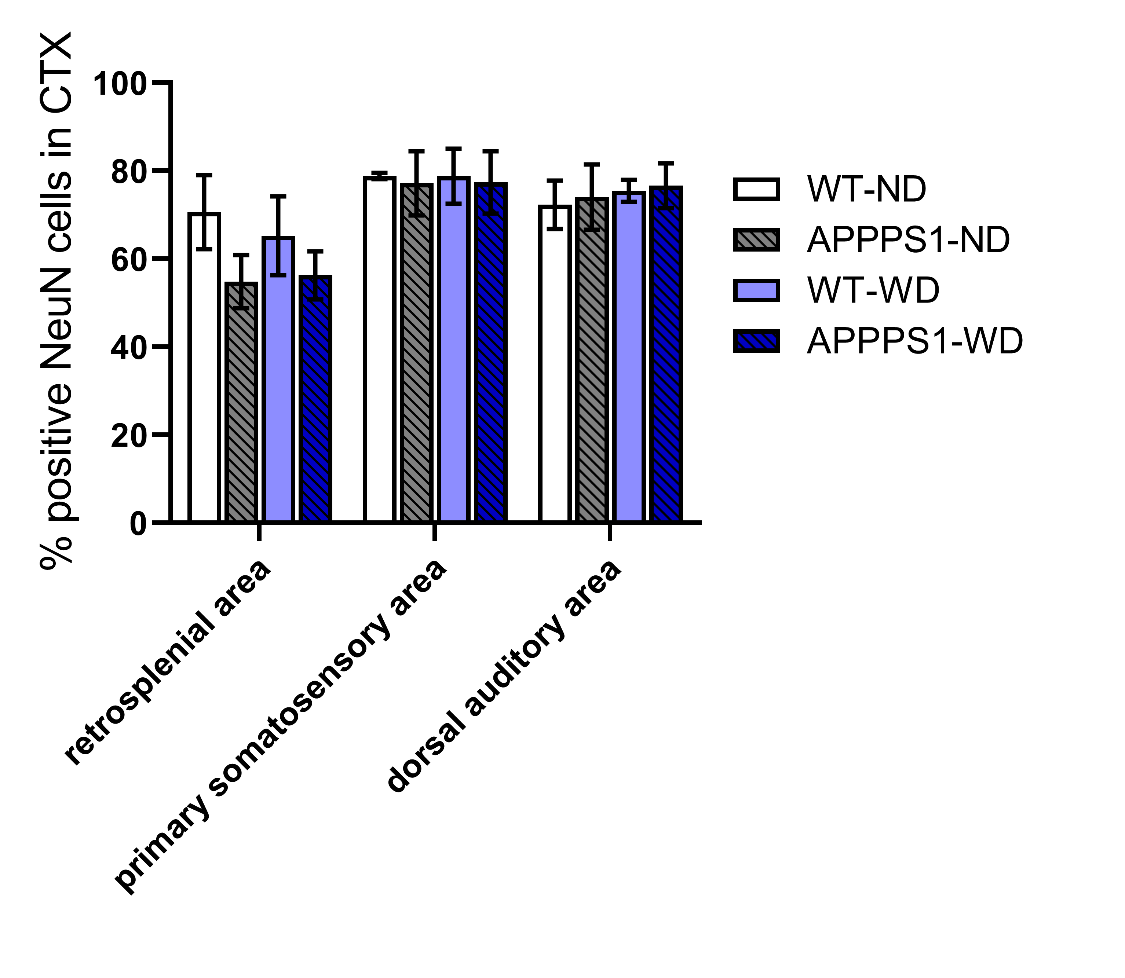


Additional Figure 5:

Percentage of positive NeuN cells in cortex (CTX) areas: retrosplenial area, primary somatosensory area and dorsal auditory area. WT-ND *n* = 2; APPPS1-ND *n* = 3; WT-WD *n* = 3; APPPS1-WD *n* =3.
